# Sequential Delivery of Novel Triple Drug Combination via Crosslinked Alginate/Lactoferrin Nanohybrids for Enhanced Breast Cancer Treatment

**DOI:** 10.3390/pharmaceutics14112404

**Published:** 2022-11-08

**Authors:** Mai Salah, Marwa A. Sallam, Mona A. Abdelmoneem, Mohamed Teleb, Kadria A. Elkhodairy, Adnan A. Bekhit, Asmaa F. Khafaga, Ahmed E. Noreldin, Ahmed O. Elzoghby, Sherine N. Khattab

**Affiliations:** 1 Cancer Nanotechnology Research Laboratory (CNRL), Faculty of Pharmacy, Alexandria University, Alexandria 21521, Egypt; 2 Department of Industrial Pharmacy, Faculty of Pharmacy, Alexandria University, Alexandria 21521, Egypt; 3 Department of Pharmaceutics, Faculty of Pharmacy, Damanhour University, Damanhour 22511, Egypt; 4 Department of Pharmaceutical Chemistry, Faculty of Pharmacy, Alexandria University, Alexandria 21521, Egypt; 5 Pharmacy Program, Allied Health Department, College of Health Sciences, University of Bahrain, Sakheer P.O. Box 32 038, Bahrain; 6 Department of Pathology, Faculty of Veterinary Medicine, Alexandria University, Edfina 22758, Egypt; 7 Department of Histology and Cytology, Faculty of Veterinary Medicine, Damanhour University, Damanhour 22511, Egypt; 8 Chemistry Department, Faculty of Science, Alexandria University, Alexandria 21321, Egypt

**Keywords:** protein/polysaccharide nanohybrids, sodium alginate, lactoferrin, rosuvastatin, pemetrexed, honokiol, breast cancer treatment

## Abstract

While breast cancer remains a global health concern, the elaboration of rationally designed drug combinations coupled with advanced biocompatible delivery systems offers new promising treatment venues. Herein, we repurposed rosuvastatin (RST) based on its selective tumor apoptotic effect and combined it with the antimetabolite pemetrexed (PMT) and the tumor-sensitizing polyphenol honokiol (HK). This synergistic three-drug combination was incorporated into protein polysaccharide nanohybrids fabricated by utilizing sodium alginate (ALG) and lactoferrin (LF), inspired by the stealth property of the former and the cancer cell targeting capability of the latter. ALG was conjugated to PMT and then coupled with LF which was conjugated to RST, forming core shell nanohybrids into which HK was physically loaded, followed by cross linking using genipin. The crosslinked HK-loaded PMT–ALG/LF–RST nanohybrids exhibited a fair drug loading of 7.86, 5.24 and 6.11% for RST, PMT and HK, respectively. It demonstrated an eight-fold decrease in the IC50 compared to the free drug combination, in addition to showing an enhanced cellular uptake by MCF-7 cells. The in vivo antitumor efficacy in a breast cancer-bearing mouse model confirmed the superiority of the triple cocktail-loaded nanohybrids. Conclusively, our rationally designed triple drug-loaded protein/polysaccharide nanohybrids offer a promising, biocompatible approach for an effective breast tumor suppression.

## 1. Introduction

Breast cancer is the second most common cause of death in women after lung cancer [1]. Many reports have suggested that by 2050, breast cancer will spread to reach approximately 3.2 million new cases [2]. Extensive efforts are continuously ongoing to develop new chemotherapeutics for clinical purposes. While traditional anticancer treatment remains a major clinical concern, new strategies are now being implemented to overcome the main restrictions of chemotherapy, ranging from random biodistribution to systemic toxicity [3]. In that context, numerous nanoparticle (NP)-based delivery systems have been developed to deliver single or combined anticancer agents. The use of a combination strategy represents a successful approach for chemotherapy treatment [4]. To overcome multidrug resistance (MDR) and enhance drug effectiveness against both drug-resistant and drug-sensitive cancer cells by enhancing chemosensitivity and drug bioaccessibility, some nanodrug delivery systems have been developed by combining nanotechnology with multidrug chemosensitization [5]. Pemetrexed (PMT) is an active multidirectional antifolate cytotoxic chemotherapeutic drug against various kinds of cancer, such as breast cancer [6]. However, the clinical benefits are limited due to its poor therapeutic results related to the inability to achieve sufficient intracellular concentrations at the doses limit allowed, while increasing the PMT dose results in systemic toxicity and MDR. PMT also has low bioavailability and selectivity [7].

Accordingly, several attempts have been made to enhance the antitumor effect of PMT, such as combining it with other chemotherapeutic agents or phytomedicines [8,9]. In addition, the bioavailability of PMT has been improved, and side effects have been reduced by various targeted drug delivery systems through the coupling of drugs with enhancer peptides or nanoformulations [10].

Rosuvastatin (RST) is one of the statins used to treat hypercholesterolemia [11,12]. It mediates its action by suppressing β-hydroxy β-methylglutaryl-CoA (HMG-CO A) reductase and blocking the mevalonate (MVA) pathway. The suppression of the MVA pathway in addition to reducing cholesterol synthesis has been reported to induce selective tumor cell apoptosis events [13]. Moreover, HK, which is a herbal extract of *Magnolia grandiflora* seeds, has attracted much attention by virtue of its advantageous antitumor actions.

The combination of PMT, RST and HK were proposed based on the predicted synergy between the three drugs due to their multiple mechanistic pathways (Figure S1 and Supporting Information). RST blocks the MVA pathway and thus triggers the activation of the sterol regulatory element-binding protein 2 (SREBP2) as a restorative feedback response to restore homeostasis. On the other hand, SREBP2 processing can be blocked by HK. Accordingly, HK enhances the ability of RST to activate the apoptosis of cancer cells [14]. RST also could synergize with the apoptotic action of PMT, enhancing its cytotoxicity through the inhibition of the Ras-Raf-1-MAPK signaling pathway [15]. Additionally, HK enhances the efficiency of PMT by minimizing the occurrence of MDR as HK suppresses the P-glycoprotein efflux pumps to make drug-resistant tumor cells sensitive to chemotherapeutic drugs [16].

The delivery of chemotherapeutics with nanosized polymeric carriers offers various advantages, including an efficient drug loading, a targeted release and an enhanced accumulation of drugs in tumor cells, which reduces the side effects, in addition to enhancing the circulation times and providing a better bioavailability [17]. Among nanocarriers, protein- and polysaccharide-based NPs have many advantages, such as biodegradability, biocompatibility, an ease of functionalization, negligeable toxicity profiles and an enhanced biodistribution [17,18,19,20]. Natural proteins and polysaccharides can be utilized in chemical coupling because they have many reactive functional groups, including carboxylic, thiol and amino groups. Accordingly, we combined the advantages of protein polysaccharide nanohybrids and polymeric drug conjugates to elaborate an injectable platform for hydrophobic anticancer agents, ensuring stability in the bloodstream and allowing for a release at the tumor site [21].

The NP shell requires stealth properties to prevent the reticuloendothelial recognition and a subsequent removal, thus allowing for a passive accumulation in the tumor cells. Polysaccharides, such as sodium alginate (ALG), can provide stealth properties for nanoparticles and reduce the plasma protein adsorption [22]. ALG is a hydrophilic salt of alginic acid that is a nontoxic, natural polysaccharide that exists in all types of brown algae. Additionally, ALG is a biodegradable polymer employed extensively in the medical, food and pharmaceutical industries. Previous studies have reported many applications of ALG for drug delivery, and it has been used to prepare the sustained release delivery systems for various drugs [23].

Furthermore, lactoferrin (LF), a member of the transferrin family, has antioxidant, anti-inflammatory, immunostimulatory and established anticancer effects. The internalization of LF into cancer cells has been reported due to its great affinity to bind with many receptors overexpressed on the surface of tumor cells, such as LF receptors (LRP1, LRP2) and low-density lipoprotein (LDL). Moreover, the nuclear localization sequence of LF enables it to be internalized into the nucleus and the site of action of most chemotherapeutics, and it is reported to target the delivery of cytotoxic agents to tumor cells [24]. In our study, LF was utilized as a nanocarrier to improve the loading capacity, antitumor efficacy and solubility of the hydrophobic drug RST.

Herein, we propose crosslinked ALG/LF NHs to deliver PMT, RST and HK for breast cancer treatment. **First**, PMT, a highly soluble chemotherapeutic drug, has been conjugated to ALG to attenuate its release into the systemic circulation, therefore minimizing the side effects and enhancing the accumulation within tumor cells. **Second**, RST was conjugated to the LF polymer to provide a sustained release pattern of RST and increase its solubility. **Third**, protein-polysaccharide (PMT–ALG/LF–RST) NHs were developed by chemical coupling. **Fourth**, HK, which is a hydrophobic drug, has been incorporated into the hydrophobic core of nanohybrids to overcome its solubility problem. **Finally**, the crosslinking of HK-loaded PMT–ALG/LF–RST NHs with genipin was performed to enhance the stability of its structure, sustain the drug release and prohibit its premature disintegration. The developed nanohybrids demonstrated greater antitumor effects than the free drug combination using in vitro and in vivo investigations.

## 2. Materials and Methods

### 2.1. Materials

The Supplementary File includes all the chemicals utilized in this research.

### 2.2. Preparation of Crosslinked HK-Loaded PMT–ALG/LF–RST NHs F10

#### 2.2.1. Preparation of Alginate/Lactoferrin Nanohybrids (ALG/LF NHs) **F1**

The nanohybrids were fabricated through a carbodiimide coupling reaction between the carboxyl groups of the ALG and LF amino groups. Sodium alginate (0.05 g) was dissolved in 7 mL of double filtrated distilled H_2_O. The preactivation of the carboxylic acid groups of ALG was performed for 5 min by the in situ addition of (0.01 g, 0.05 mmol) EDC. The HCl and (0.009 g, 0.05 mmol) K. Oxyma at room temperature (RT) were under a constant stirring for 5 min. An aqueous solution of (0.10 g, 0.00125 mmol) lactoferrin (5 mL) was added dropwise to the reaction mixture. The reaction mixture was stirred overnight. Dialysis was performed on the resulting solution against double filtrated distilled H_2_O for 48 h to eliminate the byproducts, and the lyophilization of the product was performed for a further investigation.

#### 2.2.2. Preparation of Lactoferrin–Rosuvastatin Conjugate (LF–RST) **F2**

First, the addition of DIC (0.019 mL, 0.12 mmol) and Oxyma (0.017 g, 0.12 mmol) to (0.025 g, 0.025 mmol) the calcium salt of the RST solution in 5 mL of DMSO was carried out. Then, the RST was preactivated at RT for 10 min to completely convert the carboxylate group to the corresponding active ester. (0.10 g, 0.00125 mmol) Lactoferrin dissolved in DMSO (5 mL) was added dropwise to the reaction mixture and stirred overnight at RT. Dialysis was performed on the resulting solution against DMSO for 24 h, and the dialysate was collected to indirectly determine the RST content. Then, the dialysis of the reaction mixture against the double filtrated distilled H_2_O was carried out by gradually changing the DMSO and H_2_O ratio (90:10, 70:30, 50:50, 30:70 and 10:90) for 72 h. A further dialysis of the reaction mixture was carried out against the double filtrated distilled H_2_O, followed by the lyophilization.

#### 2.2.3. Preparation of Alginate/Lactoferrin–Rosuvastatin Nanohybrids Loaded with Honokiol (HK-Loaded ALG/LF–RST NHs) **F4**

Sodium alginate (ALG, 0.05 g) was dissolved in 7 mL of double filtrated distilled H_2_O. The activation of the carboxylic group of ALG was carried out by the in situ addition of 0.009 g (0.05 mmol) of K. Oxyma and 0.01 g of (0.05 mmol) EDC. The HCl at RT was under constant stirring for 5 min. The prepared aqueous solution of LF–RST conjugate (12 mL, 0.116 g, Section 2.2.2) was then added dropwise to the activated ALG solution. The reaction was stirred at RT for 24 h. The resultant nanohybrids were dialyzed against double filtrated distilled water to obtain the ALG/LF–RST NHs **F3**. The solvent evaporation method was adopted for the physical loading of HK into the core of the ALG-LF–RST NHs. An amount of 0.015 g of HK was dissolved in 0.3 mL of ethyl alcohol, added to ALG/LF–RST NH colloidal solution, and stirred overnight to allow for the slow evaporation of the organic solvent and physical loading of the drug within the hydrophobic core of the nanohybrids. Finally, the product was centrifuged at 4 °C for 10 min, filtrated to eliminate the unentrapped drug, and it was lyophilized.

#### 2.2.4. Preparation of Pemetrexed–Alginate Conjugate (PMT–ALG) **F5**

K. Oxyma (0.018 g, 0.1 mmol) and EDC. HCl (0.02 g, 0.1 mmol) was added to (0.015 g, 0.035 mmol) the PMT solution in 5 mL of double filtrated distilled H_2_O. Then, the addition of the prepared ALG solution (0.05 g of ALG dissolved in 7 mL double filtrated distilled H_2_O) to the reaction mixture was performed and stirred overnight at RT. The reaction mixture was dialyzed against the double filtrated distilled H_2_O for 24 h. Then, the dialysate was collected to determine the unconjugated PMT, followed by the successive replacement of distilled H_2_O for an additional 48 h, followed by lyophilization.

#### 2.2.5. Preparation of Pemetrexed–Alginate/Lactoferrin Nanohybrids Loaded with Honokiol (HK-Loaded PMT–ALG/LF NHs) **F7**

The PMT–ALG conjugate aqueous solution (13 mL, 0.062 g, prepared as described in Section 2.2.4) was further preactivated for 5 min using (0.01 g, 0.05 mmol) EDC.HCl and 0.009 g (0.05 mmol) of K. Oxyma at RT under a constant stirring. An aqueous solution (5 mL) of lactoferrin (0.10 g, 0.00125 mmol) was added dropwise to the reaction mixture, which was stirred for 24 h at RT. The resultant PMT–ALG/LF NHs **F6** were then purified by dialysis against double filtrated distilled water. The solvent evaporation method was adopted for the physical loading of 0.015 g of HK in 0.3 mL of ethanol into the core of PMT–ALG/LF nanohybrids, as presented previously in Section 2.2.3. The newly prepared NHs were lyophilized for a further characterization.

#### 2.2.6. Preparation of Pemetrexed–Alginate/Lactoferrin–Rosuvastatin Nanohybrids (PMT–ALG/LF–RST NHs) **F8**

The PMT–ALG conjugate aqueous solution (13 mL, 0.062 g, prepared as described in Section 2.2.4) was preactivated for 5 min by (0.01 g, 0.05 mmol) EDC. The HCl and 0.009 g (0.05 mmol) of K. Oxyma at RT were under a constant stirring. The aqueous solution of the LF–RST conjugate (12 mL, 0.116 g, prepared as given in Section 2.2.2) was then added dropwise to the reaction mixture and stirred overnight at RT. The resultant nanohybrids were then dialyzed against the double filtrated distilled H_2_O and lyophilized for a further characterization.

#### 2.2.7. Physical Loading of HK within PMT–ALG-LF–RST Nanohybrids (HK-Loaded PMT–ALG/LF–RST NHs) **F9**

The solvent evaporation method was used to load HK into the core of the PMT–ALG/LF–RST NHs **F8**. An amount of 0.015 g of HK dissolved in 0.3 mL of ethanol was added to 27 mL (0.178 g equivalent to 0.016 g RST, 0.012 g PMT and 0.014 g HK) of the prepared PMT–ALG/LF–RST NHs **F8**. The resulting colloidal solution was treated as described previously in Section 2.2.3, followed by the lyophilization.

#### 2.2.8. Crosslinking of HK-Loaded PMT–ALG/LF–RST Nanohybrids (Crosslinked HK-Loaded PMT–ALG/LF–RST NHs) **F10**

Genipin (0.035 g, 0.155 mmol) was added to 22 mL (0.192 g equivalent to 0.016 g RST, 0.012 g PMT and 0.014 g HK) of the prepared HK/PMT–ALG/LF–RST NHs **F9** and left for 48 h under a constant stirring to achieve particle crosslinking. The prepared colloidal crosslinked HK/PMT–ALG/LF–RST NHs **F10** were freeze dried to obtain a blue powder.

### 2.3. Physicochemical Characterization of Crosslinked HK-Loaded PMT–ALG/LF–RST NHs

Many methods have been performed to estimate the physicochemical characteristics of the synthesized nanohybrids. The loading and conjugation of drugs were studied via DSC, HPLC, FT-IR and ^1^H-NMR spectroscopy. The release of drugs was investigated via the dialysis membrane method and HPLC, while the zeta potential and particle size were measured utilizing a Malvern Zetasizer, and the particle morphology was investigated by TEM. Additionally, the dispersibility and stability of the synthesized nanohybrids were investigated thoroughly. In addition, the lyophilization, redispersibility, physical stability testing and in vitro hemolysis and serum stability were performed as detailed in the Supporting Information (Sections S1.3–S1.11).

### 2.4. In Vitro Cytotoxicity and Cellular Uptake Study

The cytotoxicity of free HK, free RST, free PMT, free PMT/RST combination, free PMT/HK combination, free HK/RST combination, free RST/PMT/HK combination, HK-loaded ALG/LF–RST NHs **F4**, HK-loaded PMT–ALG/LF NHs **F7**, PMT–ALG/LF–RST NHs **F8**, HK-loaded PMT–ALG/LF–RST NHs **F9** and crosslinked HK-loaded PMT–ALG/LF–RST NHs **F10** on MCF-7 breast cancer cells was investigated by the MTT assay detailed in the Supporting Information (Section S2). The intensity of the cellular uptake was compared to uncrosslinked PMT–ALG/LF–RST and crosslinked PMT–ALG/LF–RST NHs using flow cytometry (Supporting Information (Section S3)).The cellular uptake of the uncrosslinked PMT–ALG/LF–RST and crosslinked PMT–ALG/LF–RST NHs into MCF-7 cancer cells was investigated by confocal microscopy, as described in the Supporting Information (Section S3).

### 2.5. In Vivo Antitumor Efficacy

The in vivo antitumor efficacy of the crosslinked HK-loaded PMT–ALG/LF–RST NHs **F10** was compared with the free RST, free HK, free PMT and free (HK/RST/PMT) combination therapy solution using female mice in accordance with the standard protocol described in the Supporting Information (Section S4, Table S1).

## 3. Results

### 3.1. Synthesis of PMT–ALG/LF–RST NHs ***F8***

**Figure 1:**
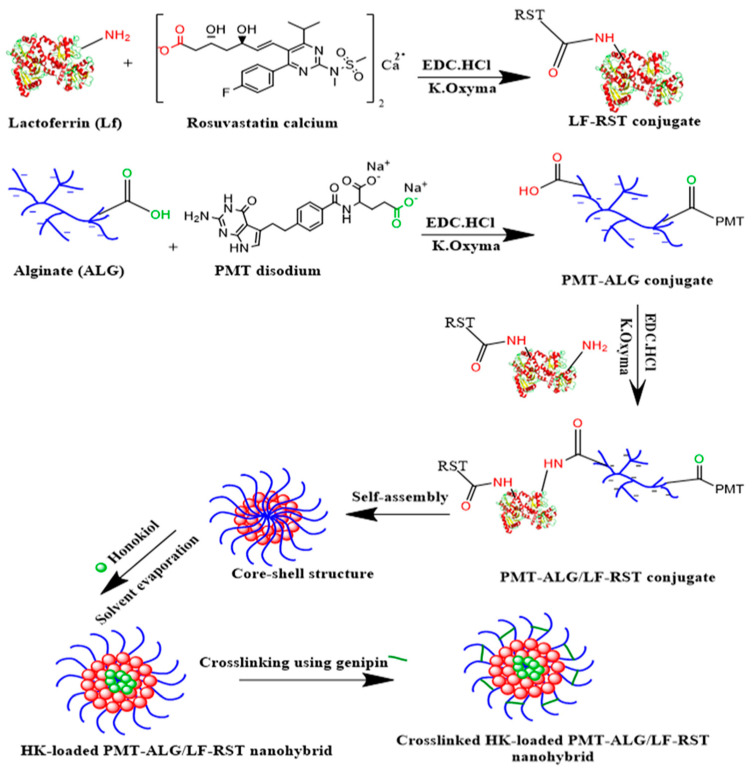
Schematic diagram showing the formulation stages of crosslinked HK-loaded PMT–ALG/LF–RST NH **F10**.

In this research, novel ALG/LF NHs were developed via chemical conjugation for the delivery of combined poorly soluble anticancer drugs, such as RST and HK, and the highly soluble cytotoxic drug PMT to breast cancer cells. ALG is a hydrophilic polysaccharide that exists in all types of brown algae. In addition to the ALG biodegradability and biocompatibility, the rationale for choosing ALG as a nanoparticle shell is to exploit its stealth property to prevent the reticuloendothelial recognition and subsequent removal, thus allowing for a passive accumulation of the nanoparticles in the tumor. Furthermore, lactoferrin (LF), a member of the Tf family, has established anticancer properties. Moreover, the rationale for choosing LF was to exploit its selective tumor-targeting action by binding to the overexpressed multiple receptors in breast tumor cells. The conjugation of chemotherapeutic agents such as PMT and RST to ALG and LF, respectively, can enhance their efficacy and bioavailability and reduce the side effects. Thus, the RST conjugation to LF would improve its water solubility due to the hydrophilic nature of LF. Additionally, the PMT conjugation to ALG would sustain its release in the systemic circulation, hence enabling its targeted delivery into tumor cells. In the current study, PMT–ALG/LF–RST NHs were developed through three steps. First, the LF–RST conjugate was synthesized via the formation of an amide bond via carbodiimide coupling. DIC/Oxyma was used to activate the carboxylic acid side chains of RST; thus, an intermediate Oxyma-activated ester molecule was formed and covalently coupled with the amine groups of LF [25,26,27,28,29,30,31]. The LF–RST conjugate showed a particle size of 179.0 nm with a high RST loading (15.25 ± 0.56 wt.%) and ζ-potential of +14.7 mV. This is similar to a previous study reported by Abdelmoneem et al., which fabricated an LF–Celastrol (LF–CST) conjugate, where the hydrophilic property of LF was utilized to solubilize the hydrophobic drug [24]. Second, the PMT–ALG conjugates were synthesized through ester bond formation by carbodiimide coupling. EDC.HCl/K. Oxyma was used to activate the carboxylic acid side chains of PMT to covalently couple with the hydroxyl groups of ALG. The resulting PMT–ALG conjugate showed a particle size of 267.9 nm with a high PMT loading (19.35 ± 0.64 wt.%) and ζ-potential of −47.1 mV. Recently, polysaccharide drug conjugation was reported by Zhou et al., where dextran–RST was also prepared by a carbodiimide coupling reaction [32]. Third, PMT–ALG/LF–RST NHs were fabricated by coupling the PMT–ALG conjugate to the LF–RST conjugate through the formation of an amide bond by utilizing coupling reagents such as K. Oxyma and EDC. HCL, which enables the covalent coupling of the free carboxylic acids of the PMT–ALG, conjugates with the free amino groups of the LF–RST conjugate. These newly synthesized nanohybrids can self-assemble into spherical nanohybrids consisting of an LF–RST conjugate inner core as a reservoir for hydrophobic drugs and a PMT–ALG conjugate as a hydrophilic shell, where the conjugation of RST to the LF polymer could also increase the hydrophobicity of LF [33,34]. In our preliminary study, two conjugation ratios between ALG and LF were investigated for the preparation of the ALG/LF NHs (Table 1). The ALG:LF (1:2) ratio was finally selected based on the zeta potential and particle size characterization. The resultant PMT–ALG/LF–RST NHs exhibited a particle size of 304.9 nm and a greatly negative ζ-potential of −43.8 mV, which might correspond to the free COOH groups of ALG on the surface of the copolymer (Table 1). The preparation steps of the crosslinked HK-loaded PMT–ALG/LF–RST NHs are illustrated in the schematic diagram shown in Figure 1.

**Figure 2:**
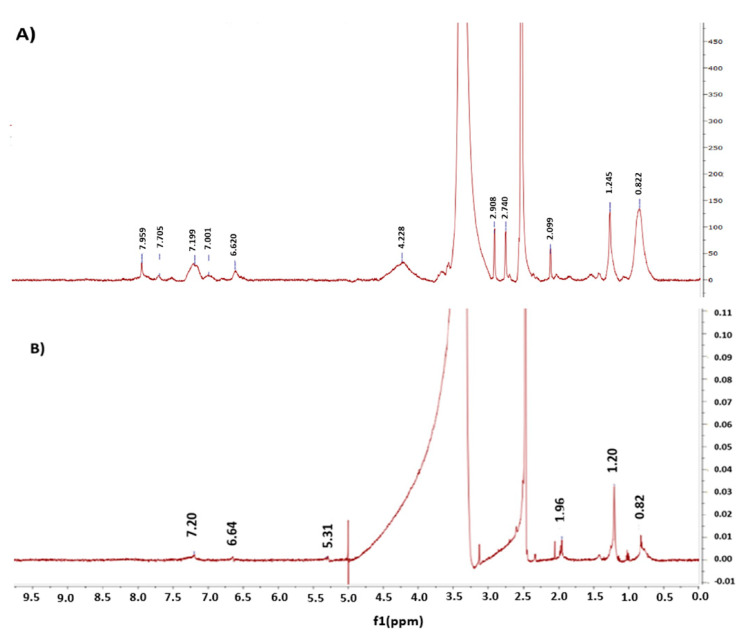

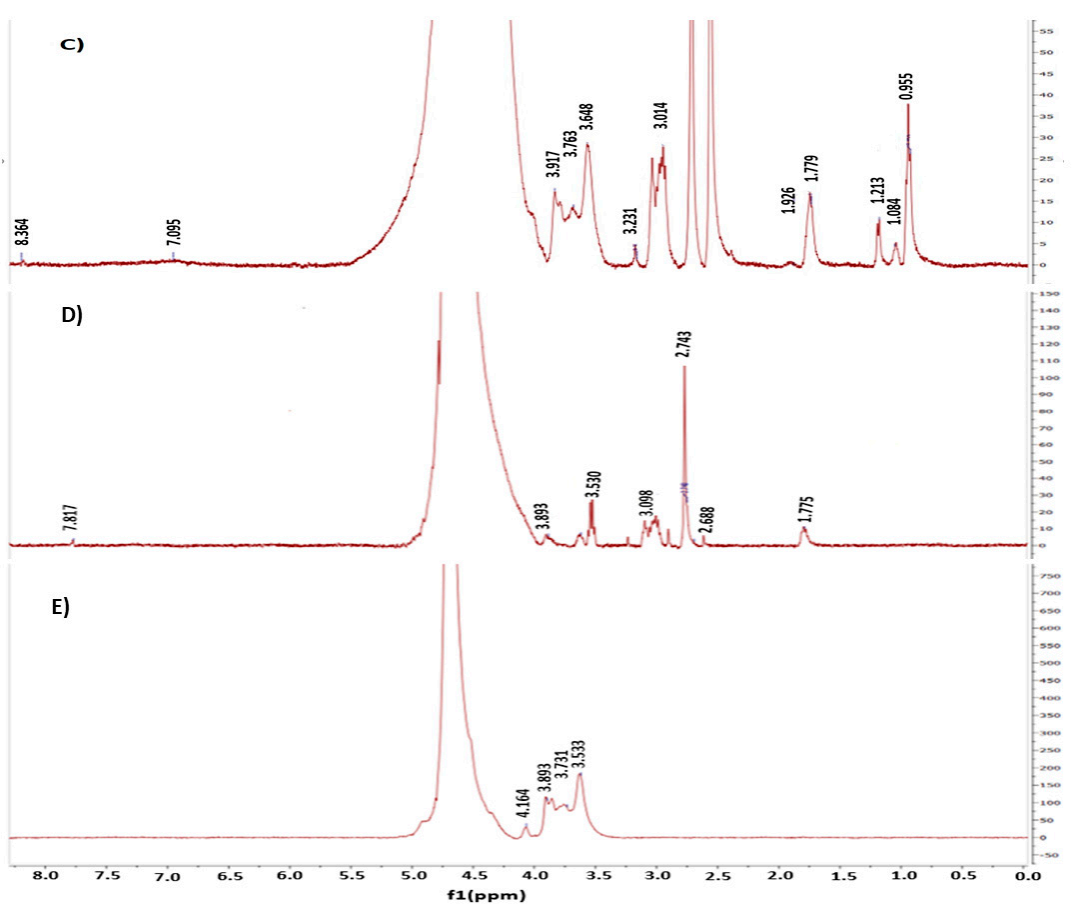
^1^H NMR (DMSO-*d_6_*) spectra of (**A**): LF–RST, (**B**): LF and ^1^H NMR (D_2_O) spectra of (**C**): PMT–ALG/LF–RST NHs, (**D**): PMT–ALG and (**E**): ALG.

The conjugation reactions were confirmed by ^1^H-NMR analysis (Figure 2). The ^1^H-NMR spectrum of the LF–RST conjugate (Figure 2A) reveals a broad singlet peak at 1.25 ppm attributed to the methyl groups of RST. In addition, two singlet peaks at 2.74 and 2.91 ppm corresponding to the S–CH_3_ and N–CH_3_ groups of RST, respectively, were observed. Additionally, multiplet peaks attributed to the aromatic protons of RST were observed in the range of 6.90–8.00 ppm. The ^1^H-NMR spectrum of LF in DMSO-*d_6_* (Figure 2B) shows multiplet peaks at the variation between 0.50 and 2.40 ppm which are attributed to the peptide chain aliphatic protons of LF. Moreover, two broad multiplet peaks were observed at 6.60–6.70 and 7.10–7.30 ppm, attributed to the peptide chain aromatic protons and NH protons. On the other hand, the ^1^H-NMR spectrum of the PMT–ALG–LF–RST NHs (Figure 2C) shows three multiplet peaks in the range of 0.80–1.23 ppm attributed to the aliphatic protons of the LF–RST conjugate. Furthermore, peaks in the range of 1.70–3.10 ppm were observed to be characteristic of the methylene protons of PMT. Moreover, multiplet peaks attributed to the alginate aliphatic C–H protons were observed in the range of 3.64–4.00 ppm. The ^1^H-NMR spectrum of the ALG–PMT conjugate in D_2_O (Figure 2D) shows peaks in the range of 1.70–3.10 ppm, characteristic of the methylene protons of PMT. Moreover, multiplet peaks were observed in the range of 7.81–7.82 ppm related to the aromatic protons of PMT. The ^1^H-NMR spectrum of ALG in D_2_O (Figure 2E) shows multiplet peaks in the range 3.64–4.16 ppm.

### 3.2. Development of Crosslinked HK-Loaded PMT–ALG/LF–RST NHs

**Figure 3:**
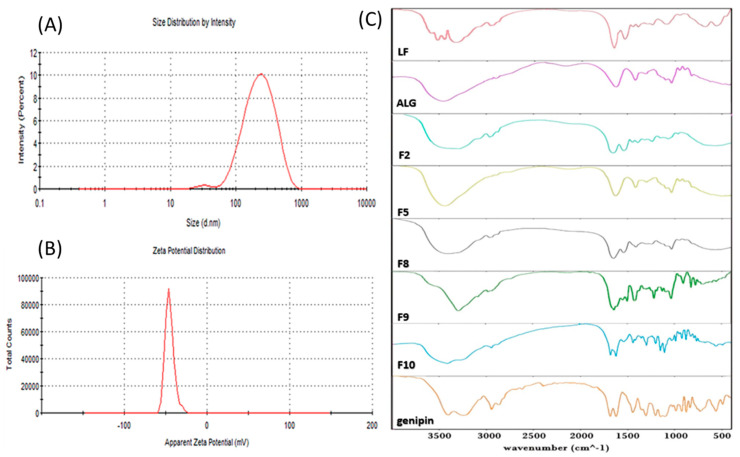
(**A**) Size distribution diagram of crosslinked HK-loaded PMT–ALG/LF–RST NHs **F10**; (**B**) ζ potential of NHs **F10** and (**C**) Fourier transform infrared (FTIR) spectra of LF, ALG, LF–RST **F2**, PMT–ALG F5, PMT–ALG/LF–RST **F8**, HK-loaded PMT–ALG/LF–RST **F9**, genipin and crosslinked HK-loaded PMT–ALG/LF–RST NHs **F10**.

In contrast to PMT and RST, which were covalently coupled to the ALG–LF backbone, HK was physically loaded inside the hydrophobic core of the PMT–ALG/LF–RST NHs via a simple solvent evaporation method. There may be an abundance of co-acting intermolecular interactions between the carrier material and the drug in the loading process, such as van der Waal forces, hydrophobic interactions and hydrogen bonding. All of these forces can play a role in effective HK loading and nanohybrid stabilization [35,36]. Finally, the crosslinking of the polymeric nanohybrids with genipin seems to be an excellent strategy to enhance their structural stability and prevent a rapid drug release and premature disintegration. This study revealed that genipin successfully crosslinked the amine groups of LF with a significant reduction in the nanohybrid size and drug release profile. The crosslinking reaction by genipin led to the appearance of an intense blue color. Upon the crosslinking of the nanohybrids, the particle size markedly decreased from 389 nm to 258.7 nm by virtue of forming more compact and denser nanohybrids (Table 1 and Figure 3A,B) [1]. During our preliminary investigations, different amounts of genipin were used for the crosslinking of ALG–LF nanohybrids (Table 2). Approximately 35 mg (1:5.54 wt. ratio) of genipin was finally selected based on the PS and PDI characterization. 

### 3.3. Solid-State Characterization

The FT-IR spectra of our prepared formulations were used to study the chemical modification (Figure 3C). The FT-IR spectrum of the LF–RST conjugate shows two bands at 1317 and 1151 cm^−1^ which are attributed to the SO_2_ group of RST. Additionally, a band at 1540 cm^−1^ specific to the C=N group of RST was observed. Furthermore, the LF characteristic absorption bands ranging between 3600 and 2600 cm^−1^ are assigned to the N–H and hydroxyl groups, where bands at 2961 cm^−1^ and 2865 cm^−1^ are assigned to the sp^3^ C–H stretching, where vibrations were observed. Additionally, the most distinctive bands of LF at 1651 (amide I) and 1448 (amide II) cm^−1^ were observed. The new amidic carbonyl group in the LF–RST conjugate overlapped with that of LF at 1651 cm^−1^, which confirms an amide bond formation between LF and RST. On the other hand, the FT-IR spectrum of the ALG–PMT conjugate (Figure 3C) showed a broad stretching band in the range from 3300 to 3000 cm^−1^ which is attributed to the hydroxyl group of ALG. Furthermore, the disappearance of the strong band of PMT at 1690 cm^−1^ and the broad band ranging between 3600 and 2500 cm^−1^ attributed to carboxylic acid confirms the PMT conjugation. Moreover, a stretching band at 1701 cm^−1^ related to the new ester carbonyl group in the PMT–ALG conjugate was observed [33]. In addition, the FT-IR spectrum of the PMT–ALG-LF–RST NHs (Figure 3C) shows an absorption band at 1652 cm^−1^, corresponding to the new amide carbonyl group of the nanohybrids overlapped with that of the LF and LF–RST conjugates at 1651 cm^−1^. Moreover, an absorption band at 1302 cm^−1^ attributed to the SO_2_ group of RST was observed. The band at 1542 cm^−1^ related to the C=N group of RST was also noticed. In addition, the FT-IR spectrum of the HK-loaded PMT–ALG/LF–RST NHs reveals a broad absorption band at 3292 cm^−1^, which is related to the OH group of HK. This band overlapped with the broad band between 3600 and 2500 cm^−1^, corresponding to the N–H and hydroxyl groups which are characteristic of LF and the hydroxyl group corresponding to ALG. Moreover, two absorption bands at 1639 cm^−1^ and 1426 cm^−1^ assigned to the phenyl ring of HK were observed. This absorption band (1639 cm^−1^) is overlapped by the amidic carbonyl group of the copolymer at 1652 cm^−1^. Additionally, an absorption band at 3084 cm^−1^ attributed to the sp^2^ C–H stretching band of HK was observed. The absorption band at 1217 cm^−1^ related to the C–O bond of HK was also observed. The FT-IR spectrum of the crosslinked HK-loaded PMT–ALG/LF–RST NHs (Figure 3C) shows the characteristic absorption bands of genipin, which appear in the fingerprint region of the spectrum. Moreover, a very broad absorption band between 3600 and 2600 cm^−1^ was attributed to N–H and the hydroxyl groups characteristic of LF, and the hydroxyl groups of ALG were observed.

**Figure 4:**
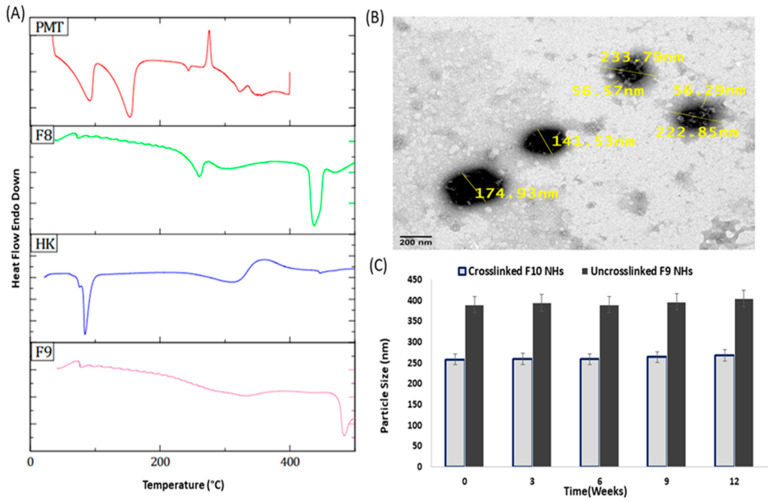
(**A**) DSC thermograms of PMT, HK, PMT–ALG/LF–RST **F8** and HK-loaded PMT–ALG/LF–RST **F9**; (**B**) TEM image of crosslinked HK-loaded PMT–ALG/LF–RST NHs **F10**; (**C**) Physical stability of uncrosslinked **F9** and crosslinked NHs **F10** revealing particle size change with time (*n* = 3).

The DSC thermograms of RST revealed endothermic peaks at approximately 80 °C and 164 °C, which were assigned to the drug melting temperature (Figure 4A) [37]. The thermogram of PMT showed three distinctive endothermic peaks at 91.78, 153.82 and 243.80 °C [38]. The characteristic peaks of RST and PMT are not observed in the PMT–ALG/LF–RST NHs **F8** thermogram, which emphasizes the amorphous nature of these NHs. Additionally, the natural state of HK exists in a crystalline form and reveals its melting peak at approximately 72.43 °C. The DSC thermogram of the HK-loaded PMT–ALG/LF–RST NHs **F9** showed only an endothermic peak at 341.29 °C, confirming the loading of HK within the NHs in an amorphous form [39]. 

### 3.4. Morphological Analysis, Physical Stability and Redispersibility

The TEM micrograph of crosslinked HK-loaded PMT–ALG/LF–RST NHs **F10** showed a spherical shape with a diameter range of 141–233 nm with no agglomerated particles, confirming their elevated colloidal stabilization (Figure 4B). The TEM images also exhibited the formation of a distinctive core–shell structure composed of the hydrophilic corona of PMT–ALG surrounding the hydrophobic core of LF–RST. It was also noticed that after being in storage for 3 months at 4 °C, both HK-loaded PMT–ALG/LF–RST NHs **F9** and crosslinked HK-loaded PMT–ALG/LF–RST NHs **F10** maintained their PSs of 410 ± 1.9 and 268 ± 0.3 nm, respectively, without a significant difference from the NHs that were initially stored, indicating their fair stability (Figure 4C). The high zeta potential (−45.3 and −44.7 mV) of both **F9** and **F10** NHs may explain their great stability, as ALG negatively charged side chains induce strong repulsive forces between the NHs. In addition to the repulsion mechanism, the stabilization of the NHs may be enhanced by the glycan chain of LF by improving the interdomain interactions of LF and protecting against the protein degradation [40,41]. The physical stability of the NHs can be further improved by the lyophilization of the prepared NHs into a dry powder [42]. In our research, no cryoprotectant was needed, and a fluffy powder was obtained that could be redispersed in H_2_O, forming a colloidal solution with no aggregation. The reconstituted lyophilized **F9** and **F10** NHs demonstrated a PS of 380 ± 0.8 and 250 ± 0.8 nm, with redispersibility index values of 0.966 and 0.975, respectively, where values less than 1.0 are considered efficient [43,44]. Furthermore, the zeta potential of the NHs after the lyophilization did not markedly change (Table 3).

### 3.5. In Vitro Drug Release

**Figure 5:**
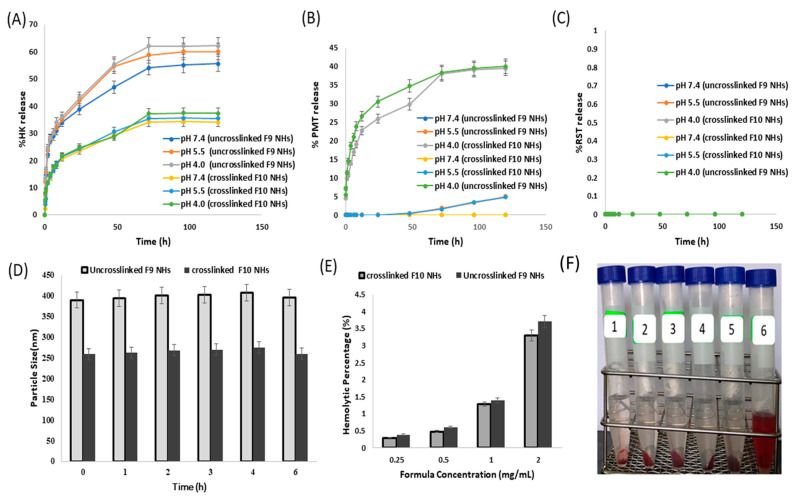
(**A**) In vitro drug release of HK from uncrosslinked **F9** and crosslinked **F10** NHs; (**B**) PMT from **F9** and **F10** NHs (*n* = 3); (**C**) RST from **F9** and **F10** NHs (*n* = 3); (**D**) serum stability of **F9** and **F10** NHs (*n* = 3); (**E**) hemolytic effect of **F9** and **F10** NHs (*n* = 3); (**F**) image of hemocompatibility at 37 °C with RBCs after 1 h of incubation.

The in vitro release of PMT, RST and HK from the uncrosslinked HK-loaded PMT–ALG/LF–RST NHs **F9** and crosslinked HK-loaded PMT–ALG/LF–RST NHs **F10** was evaluated at pH values of 4, 5.5 and 7.4 using the dialysis method in PBS (Figure 5A–C) [35]. The results revealed that the HK release from the NHs at pH values of 4, 5.5 and 7.4 did not differ significantly. The release profile of HK loaded in the NHs was biphasic with a fast release during the first 8 h (approximately 30% and 18.5% from the **F9** and **F10** NHs, respectively), followed by a slow release (with approximately 55.5% and 34% from the **F9** and **F10** NHs, respectively) for the remaining 120 h. An early rapid release can be ascribed to the fact that part of the drug is localized at the shell or the core–shell interface, but the slow-release phase of the drug can be due to that part of the drug being entrapped physically in the hydrophobic core of the nanohybrids [45]. Typically, the release rate of HK from the crosslinked NHs **F10** is slower than the rate from the uncrosslinked NHs **F9**, as the degree of nanostructural tortuosity was enhanced and the space between the polymer chains was reduced by the crosslinking [46]. Unlike HK, the results showed that crosslinking had no effect on the PMT release. PMT showed a sustained release from the NHs at a pH of 4, reaching 40% over 5 days as the ester bond can be hydrolyzed in an acidic medium, while the release decreased at a pH of 5.5 (approximately 5%), and no release was detected when the pH was 7.4 over the entire period of the experiment. The PMT release was very low due to the need for ester bond cleavage for enzymatic or chemical degradation. Our results are consistent with the previously mentioned in vitro drug release investigation, which was performed on the DTX–polymer conjugate, where the conjugate released approximately 15% over 20 days of DTX under physiological circumstances (pH 7.4) and slightly higher in an acidic environment [47]. On the other hand, even after a long-term incubation at an acidic pH, an RST release from the **F9** and **F10** NHs could not be detected. This is expected for RST, which was coupled by a highly stable amide bond that would only be cleavable inside the cancer cells under the effect of the endosomal enzymes. Markovsky et al. reported similar results after an extended incubation at an acidic pH, where no in vitro release of doxorubicin from PGA–paclitaxel–doxorubicin conjugate was observed [48]. The slow release of the drug from the developed NHs would enable them for a parenteral administration due to their stability at a physiological pH, allowing an improved drug accumulation and localized drug release at the site of the tumors [49]. 

### 3.6. Hemocompatibility and Serum Stability

The stabilization of intravenous nanoformulations in serum is important in their drug delivery application. Nanohybrids **F9** and **F10** showed no significant change in their particle size (from 389.7 ± 0.5 to 396 ± 1.2 nm and from 258.7 ± 0.95 to 260 ± 1.7 nm, respectively) when mixed with fetal bovine serum (FBS) (Figure 5D). This could be attributed to the hydrophilic brush-like structure of the ALG shell of the NHs that leads to a minimal protein adsorption on the NHs, in addition to the hydrophobic core protection from a biological invasion [50] and the surface passivation of nanohybrids. The elevated stability of the NHs in the serum might be due to a repulsion force between the serum proteins having negative charges and the prepared nanohybrids. After incubation for 4 h with FBS, the particle sizes of the **F9** and **F10** NHs reached 407 ± 0.2 nm and 275 ± 1.2 nm, respectively, which decreased to 396 ± 1.2 nm and 260 ± 1.7 nm after 6 h. This action might be attributed to the protein molecule association and dissociation on the NH surface through the incubation [51].

On another avenue, the hemolytic activity of the **F9** and **F10** NHs was approximately 3.7% and 3.3%, respectively, up to a 1 mg/mL concentration (Figure 5E,F). In general, the nontoxic and safe percentage of the hemolytic activity is less than 5% [52]. Our prepared NHs exhibited an acceptable hemolytic activity by virtue of the surface passivation of the NHs by the incorporation of the ALG polymer to suppress the protein and create a cell attachment to the surface of the NHs [53]. These results indicated that **F9** and **F10** NHs have a good hemocompatibility and are suitable for a parenteral administration.

### 3.7. In Vitro Cytotoxicity

**Figure 6:**
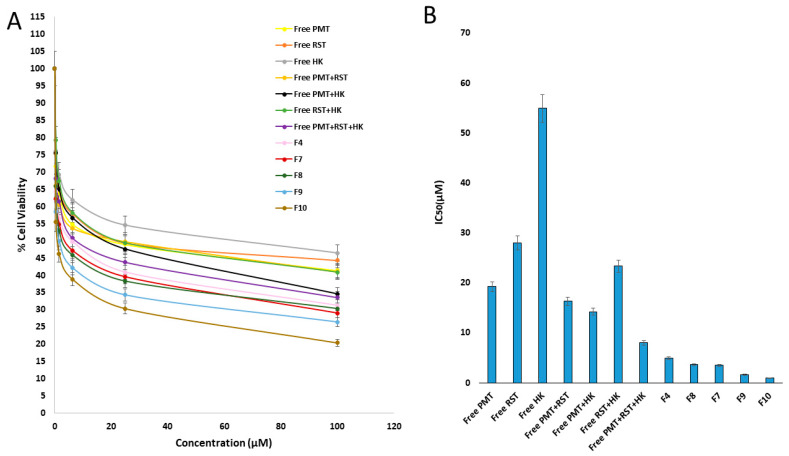
(**A**) Cytotoxicity of free HK, free RST, free PMT, free PMT/RST combination, free PMT/HK combination, free HK/RST combination, free RST/PMT/HK combination, HK-loaded ALG/LF–RST NHs **F4**, HK-loaded PMT–ALG/LF NHs **F7**, PMT–ALG/LF–RST NHs **F8**, uncrosslinked **F9** NHs and crosslinked **F10** NHs on MCF-7 breast cancer cell line. (**B**) IC_50_ of free drugs and different nanoformulations (*n* = 3).

The efficiency of the free PMT, free RST, free HK, free dual combinations (RST/HK, PMT/RST, PMT/HK) and free triple combinations (PMT/RST/HK) against cancer was studied on MCF-7 breast cancer cells compared to the developed nanohybrids after 24 h of exposure using the MTT assay (Figure 6A,B). First, the noncytotoxicity of the blank ALG/LF NHs against the MCF-7 cells after 24 h was confirmed, indicating their safety and biocompatibility (IC_50_ = 3095.443). Compared to the free single and free dual drugs, free combination therapy (PMT/RST/HK) displayed a higher cytotoxicity, revealing the synergistic effect between the three drugs. Regarding the nanohybrids, it seemed that dual drug-loaded nanohybrids (HK loaded-ALG/LF–RST NHs **F4**, PMT–ALG/LF–RST NHs **F8** and HK loaded-PMT–ALG/LF NHs **F7**) improved the potency of the combination, showing IC_50_ values with 0.61-, 0.46- and 0.44-fold reductions compared to free combination therapy (PMT/RST/HK), respectively. On the other hand, the crosslinked HK/PMT–ALG-LF–RST NHs **F10** revealed the minimum IC_50_ value in comparison to the other prepared NHs.

The CompuSyn software, mentioned by Chou and Talalay, was utilized to perform a more extensive statistical analysis [54,55,56]. The dose reduction index (DRI) and combination index (CI) were estimated to assess the antitumor efficiency of the prepared NHs relative to the free combination therapy (Table 4). The outputs showed that, compared to the free drug combination, all the prepared NHs had a higher anticancer activity, especially the uncrosslinked and crosslinked HK-loaded PMT–ALG/LF–RST NHs **F9** and **F10**, where their Cis were 0.0556 and 0.0336, respectively, supporting the synergism accomplished by a triple loading of PMT/RST/HK in the NHs. Moreover, the dose reduction indexes (DRIs) of PMT were 51.22 and 84.84 in the **F9** and **F10** NHs, respectively. The RST DRIs were 36.85 and 61.04 in the **F9** and **F10** NHs, respectively. The DRIs of HK were 111.3 and 184.34 in the **F9** and **F10** NHs, respectively.

### 3.8. In Vitro Cellular Uptake of Nanohybrids

**Figure 7:**
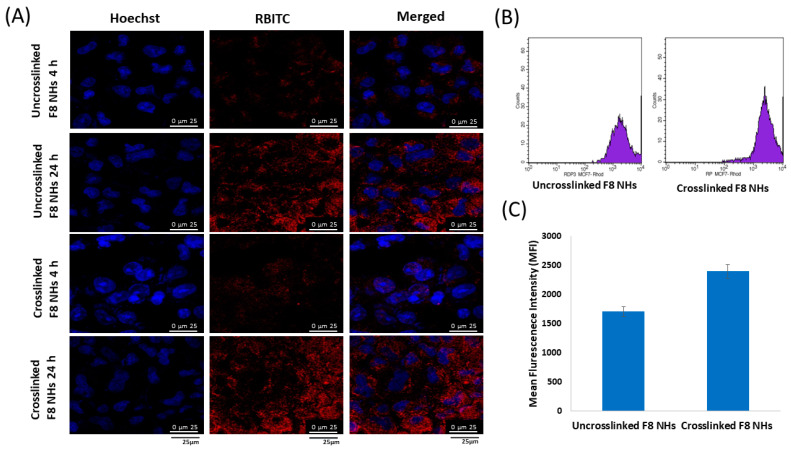
(**A**) Confocal images revealing the uptake of uncrosslinked and crosslinked PMT–ALG/LF–RST NHs **F8** after incubation for 4 h and 24 h; scale bars 25 μm; (**B**) histogram profiles of flow cytometry of MCF-7 cells after incubation for 4 h; (**C**) estimation of the mean fluorescence intensity level in MCF-7 cells after incubation for 4 h with RBITC-labeled uncrosslinked and crosslinked PMT–ALG/LF–RST NHs **F8** (*n* = 3).

For nanohybrids fluorescent labeling, the LF core of the nanohybrids was conjugated to the thiocyanate group of the RBITC dye via its free amino groups. Confocal microscopy was utilized to evaluate the uptake of the RBITC-labeled uncrosslinked PMT–ALG/LF–RST **F8** and crosslinked PMT–ALG/LF–RST NHs after incubation with MCF-7 cells at 37 °C for 4 h and 24 h (Figure 7A). Our results revealed that the crosslinked **F8** NHs exhibited a greater cellular uptake efficacy in comparison to the uncrosslinked **F8** NHs, as suggested by the powerful intensity of red fluorescence noticed in the cells treated with the former. This could be ascribed to the lower particle size of the crosslinked **F8** facilitating its cellular internalization. Those results are in accordance with previous findings by Attalah et al. who reported the enhanced cellular uptake of the crosslinked LF nanogels compared to the uncrosslinked ones [57,58]. The intensity of fluorescence for both the NHs increased after 24 h of incubation, suggesting that the process of the cellular internalization of the prepared nanohybrids is time dependent. The proton sponge effect of ALG mediates the cellular uptake of nanoparticles through enhancing the endosomal escape of nanoparticles into cytosol [57,59]. Rafiee et al. revealed that alginate nanoparticles increased the transfection rate of pEGFP in the cultured HEK 293 cells for gene delivery when compared to chitosan/alginate and Chitosan nanoparticles [60]. Flow cytometry analysis confirmed the reliability and accuracy of the results where, when comparing the fluorescent intensity of the cells treated with the uncrosslinked **F8** NHs to those treated with the crosslinked **F8** NHs, the latter indicated a much greater cellular uptake of the crosslinked **F8** after 4 h of incubation with the MCF-7 cells, as revealed in Figure 7B,C.

### 3.9. In Vivo Antitumor Efficacy

#### 3.9.1. Tumor Growth

**Figure 8:**
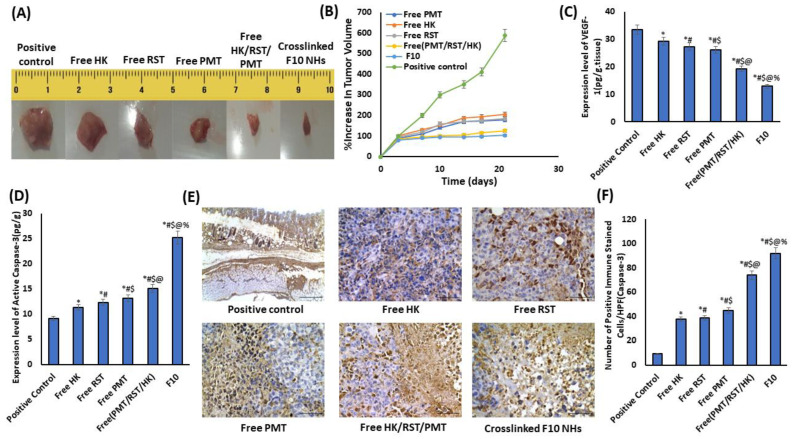
(**A**) Tumor images of positive control, free HK, free RST, free PMT, free (HK/RST/PMT) and crosslinked **F10** NHs. (**B**) Percentage of elevation in tumor volume of mice bearing EAT detected at pre-established intervals. Quantitative expression of (**C**) VEGF-1, (**D**) level of active caspase-3 for the investigated groups by ELISA. (**E**) Immunohistochemical staining of active caspase-3, and (**F**) the level of % active caspase-3 in positive control group and EAT tissues of groups treated with the free PMT, free RST, free HK, free (HK/RST/PMT) combination therapy and crosslinked HK-loaded PMT–ALG/LF–RST NHs **F10** (*n* = 4) (* *p* < 0.05 vs. positive control, # *p* < 0.05 vs. free HK, $ *p* < 0.05 vs. Free RST, @ *p* < 0.05 vs. free PMT, % *p* < 0.05 vs. free (PMT/RST/HK)).

The in vivo antitumor effect for the crosslinked HK-loaded PMT–ALG/LF–RST NHs **F10** compared with free HK, free RST, free PMT and free (HK/RST/PMT) combination treatment was investigated using mice bearing Ehrlich ascites tumors (EAT). The treatment of the mouse groups bearing EAT was conducted for three consecutive weeks while monitoring the tumor size during this period. Following treatment, the highest elevation in the tumor size percentage was in the positive control group, which reached 587%. This was higher than those detected in the free HK (205%), free RST (183%), free PMT (177%), free (HK/RST/PMT) combination therapy (125%) and crosslinked HK-loaded PMT–ALG/LF–RST-treated groups (103%) (Figure 8A,B). Obviously, the greatest anticancer activity was exhibited by the crosslinked HK-loaded PMT–ALG/LF–RST NHs **F10,** as the tumor burden was reduced in the treated mice in comparison to other groups, showing the efficacy of our rationale.

#### 3.9.2. Biomarkers of Tumor Growth

Angiogenesis plays a pivotal role in tumor metastasis and progression. The vascular endothelial growth factor (VEGF-1) is a critical factor in tumor angiogenesis. Recently, some investigations have mentioned the antiangiogenic influence of PMT, RST and HK by the downregulation of a VEGF-1 expression in the tumor cells [61,62,63,64]. Herein, ELISA was used to evaluate the degree of the VEGF-1 protein expression in the tumor tissue (Figure 8C). Using our prepared crosslinked HK loaded-PMT–ALG/LF–RST NHs **F10**, the VEGF levels were reduced successfully by 2.596-fold, while the free HK/PMT/RST combination reduced the VEGF levels only by 1.744-fold compared to the positive control.

Recent investigations have reported the apoptotic effects which are induced by HK and PMT through the upregulation of caspase-3 expression [63,65,66]. In the current investigation, the caspase 3 expression level was estimated in the tissue from EAT-bearing mice to evaluate the apoptotic effect. The results revealed that the apoptotic activity in the treated groups was greater than that in the positive control with a considerably elevated caspase-3 expression level. Our prepared crosslinked HK/PMT–ALG-LF–RST NHs **F10** succeeded in elevating the caspase-3 protein expression levels by 2.769-fold versus only a 1.659-fold increase for free HK/PMT/RST combination therapy in comparison to the positive control (Figure 8D). Moreover, the immunohistochemical investigation of the mice bearing an Ehrlich ascites tumor (EAT) confirmed our result, which revealed a marked (*p* < 0.05) increase in the count of caspase 3-positive immune stained cells in the HK-treated (37.67 ± 2.33), RST-treated (38.67 ± 1.45), PMT-treated (45.00 ± 4.04), free HK/PMT/RST combination (74.00 ± 3.21) and crosslinked HK loaded PMT–ALG/LF–RST NHs **F10** (92.00 ± 1.73) mice compared with the untreated positive control (9.33 ± 0.88) mice (Figure 8E,F).

**Figure 9:**
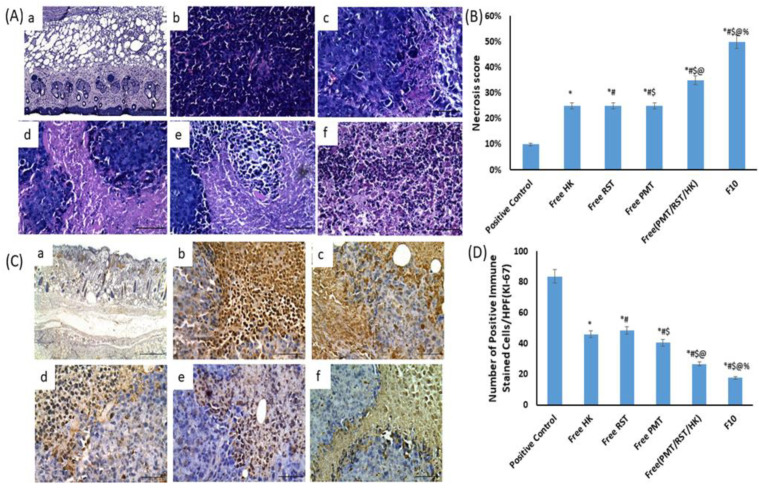
(**A**) Staining of H and E of EAT tissues of (**a**) positive control group, (**b**) free HK, (**c**) free RST, (**d**) free PMT, (**e**) free (HK/RST/PMT) combination therapy and (**f**) crosslinked HK-loaded PMT–ALG/LF–RST NHs **F10** treated groups. (**B**) The necrosis score of H and E-stained sections showed free HK-treated (approximately 25%), free RST-treated (approximately 25%), free PMT-treated (approximately 25%), free PMT/HK/RST combination (approximately 35%), and crosslinked HK-loaded PMT–ALG/LF–RST NHs **F10** (≥50%) mice compared with untreated control positive mice (approximately 10%). (**C**) Immunohistopathological staining of Ki-67 in EAT tissues. (**D**) % Ki-67 in (**a**) positive control group and EAT tissues of (**b**) free HK, (**c**) free RST, (**d**) free PMT, (**e**) free (HK/RST/PMT) combination therapy and (**f**) crosslinked HK-loaded PMT–ALG/LF–RST NHs **F10** treated groups (*n* = 4) (* *p* < 0.05 vs. positive control, # *p* < 0.05 vs. free HK, $ *p* < 0.05 vs. Free RST, @ *p* < 0.05 vs. free PMT, % *p* < 0.05 vs. free (PMT/RST/HK).

In untreated positive control mice, the solid mammary tumor showed circumscribed nodules of necrotic pleomorphic neoplastic and poorly differentiated viable cells. The viable neoplastic cells were characterized by prominent, large hyperchromatic nuclei, anisonucleosis and were bipolar to the multipolar mitotic division. However, mice treated with the free HK, free RST, free PMT, free PMT/HK/RST combination and crosslinked HK loaded-PMT–ALG/LF–RST NHs **F10** revealed a similar histologic characterization of the neoplastic cells with different degrees of necrosis (Figure 9A). Moreover, HK and PMT have been reported to enhance the death of necrotic cells in different kinds of cancer [67,68]. Necrosis scored semi-quantitatively in each excised tumor; it exhibited a significant elevation in the expression percentage in the free HK-treated (approximately 25%), free RST-treated (approximately 25%), free PMT-treated (approximately 25%), free PMT/HK/RST combination (approximately 35%) and crosslinked HK-loaded PMT–ALG/LF–RST NHs **F10** (≥50%) mice compared with the untreated control positive mice (approximately 10%) (Figure 9B). The degree of Ki-67 immunoexpression in EAT mice was evaluated to assess the proliferative activity (Figure 9C). The proliferation rate was represented by the significant (*p* < 0.05) decrease in the count of Ki67-immunoreactive cells in the HK-treated (46.00 ± 2.65), RST-treated (48.33 ± 2.33), PMT-treated (40.33 ± 2.03), free (PMT/HK/RST) combination (26.67 ± 5.04) and crosslinked HK loaded-PMT–ALG/LF–RST NHs **F10** (17.67 ± 2.33) mice compared with the untreated positive control (83.67 ± 3.38) rats (Figure 9D). PMT and RST have been reported to lower the density of the tumor cell proliferation protein Ki-67 [69,70].

#### 3.9.3. Biocompatibility and Biosafety

**Figure 10:**
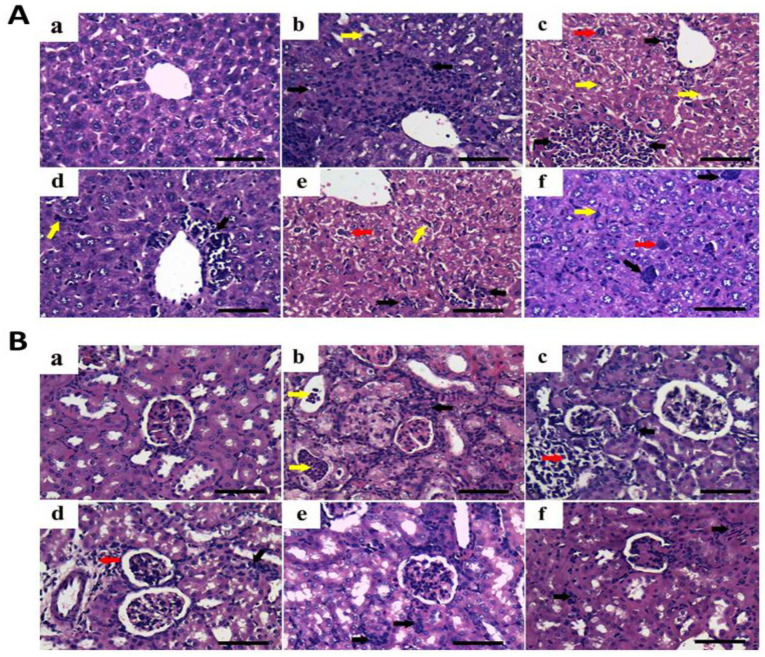
Representative photomicrographs for H&E-stained liver (**A**) and kidney (**B**) tissues (bar = 50 µm) of (**a**) positive control group, (**b**) free HK, (**c**) free RST, (**d**) free PMT, (**e**) free (HK/RST/PMT) combination therapy and (**f**) crosslinked HK-loaded PMT–ALG/LF–RST NHs **F10** treated groups. Liver: black arrows: pleomorphic, hyperchromatic, metastatic tumor foci; red arrows: chromatin condensation or abnormal mitotic figures; yellow arrows: invasion of sinusoids with tumor and Kupffer cells. Kidney: black arrows: periglomerular and interlobular infiltration; red arrows: perivascular infiltration; yellow arrows: intratubular infiltration.

The biocompatibility and biosafety of the nanohybrids was investigated in liver and kidney tissues. In liver, compared to the normal histologic picture of hepatic tissues in negative control mice, the liver tissues from untreated positive control mice showed the infiltration of pleomorphic, hyperchromatic and metastatic tumor cells in a replacement pattern; it spread out in the form of sinusoidal muralia as perivascular and interlobular multifocal foci. The replacement growth pattern tends to depend on the sinusoidal liver vasculature rather than relying on angiogenesis. The surrounding hepatic cells showed enlarged nuclei, prominent nucleoli, and the minimal degrees of chromatin condensation and abnormal mitotic figures. The activation of the Kupffer cells was also evident. However, mice treated with the free HK, free RST, free PMT, free PMT/HK/RST combination and crosslinked HK loaded-PMT–ALG/LF–RST NHs **F10** revealed a similar histopathologic characterization of the malignant cells with different degrees of invasion (Figure 10A).

In kidneys, as compared to the negative control mice, a metastatic tumorous invasion was not detected in the renal tissues of the untreated positive control mice. However, it showed a severe degree of perivascular, periglomerular and inter/or intra tubular aggregations of chronic inflammatory cells. Moreover, a moderate degree of degenerative and necrotic changes was noticed in the renal tubular epithelium. On the other hand, mice treated with the free HK, free RST, free PMT, free PMT/HK/RST combination and crosslinked HK loaded-PMT–ALG/LF–RST NHs **F10** revealed similar histopathologic details with different degrees of inflammatory infiltration, degenerative and necrotic changes (Figure 10B).

## 4. Discussion

Herein, crosslinked HK-loaded PMT–ALG/LF–RST NHs **F10** were developed to deliver a combination of poorly soluble RST and HK and highly soluble PMT anticancer drugs for a targeted breast cancer treatment. The PMT and RST drugs were conjugated to the ALG and LF polymers, respectively, by a carbodiimide conjugation reaction to form an ester bond between ALG and PMT and an amide bond between LF and RST. The conjugation of the drugs sustained an in vitro release, thus, their leakage when injected into the blood stream was prevented to avoid systemic side effects. The hydrophobic drug HK was incorporated via a physical loading within the hydrophobic core of the NHs, improving its release pattern. The crosslinking of the NHs with genipin was developed to improve the stability of the NH structure, sustain the drug release and avoid a premature disintegration. Crosslinked HK/PMT–ALG/LF–RST NHs **F10** showed a narrow PDI, an appropriate size, an elevated negative zeta potential, a high percentage drug loading of PMT, RST and HK in addition to a good serum stability and hemocompatibility. Furthermore, crosslinked HK/PMT–ALG/LF–RST NHs **F10** enhanced the cellular uptake into the MCF-7 breast cancer cell line and exhibited a superior cytotoxicity. In vivo, crosslinked HK/PMT–ALG/LF–RST NHs **F10** reduced the tumor size by inhibiting the expression levels of ki-67 and VEGF-1, which could suppress the tumor proliferation. Furthermore, the expression level of active caspase-3 was upregulated, where the induction of apoptosis in the tumor tissue of EAT-bearing mice was achieved by the crosslinked HK/PMT–ALG/LF–RST NHs **F10**. We can conclude that crosslinked HK/PMT–ALG/LF–RST NHs **F10** is a promising nanocarrier for a targeted cancer treatment.

## Figures and Tables

**Table 1:** Physicochemical characteristics and composition of crosslinked HK/PMT–ALG-LF–RST NHs. Zeta potential, particle size, entrapment efficiency (EE), drug loading (DL) and conjugation efficiency (CE %) of NPs (*n* = 3).

	Formula	Particle Size (nm)	PDI	ζ-Potential (mV)	RST		PMT		HK	
					DL mg/wt.%	%CE	DL mg/wt.%	%CE	DL mg/wt.%	%EE
**F1**	ALG/LF (1:1)	220.6 ± 1.8	0.383	−47.1 ± 0.41			-	-	-	-
**F1**	ALG/LF (1:2)	163.9 ± 2.3	0.342	−41.1 ± 0.53	-	-	-	-	-	-
**F2**	LF–RST	179.0 ± 1.3	0.371	+14.7 ± 0.91	16/13.79	64.0	-	-	-	-
**F3**	ALG/LF–RST	239.0 ± 1.8	0.348	−43.8 ± 0.27	16/9.63	64.0	-	-	-	-
**F4**	HK-loaded ALG/LF RST	365.2 ± 2.1	0.347	−46.9 ± 0.65	16/8.89	64.0	-	-	14/7.69	93.3
**F5**	ALG–PMT	267.9 ± 1.2	0.458	−47.1 ± 0.72	-	-	12/19.35	80.0	-	-
**F6**	PMT–ALG/LF	224.8 ± 1.6	0.367	−39.7 ± 0.39	-	-	12/7.40	80.0	-	-
**F7**	HK-loaded PMT–ALG/LF	333.0 ± 1.9	0.410	−41.2 ± 0.83	-	-	12/6.81	80.0	14/7.95	93.3
**F8**	PMT–ALG/LF–RST	304.9 ± 2.7	0.464	−43.8 ± 0.56	16/8.98	64.0	12/6.67	80.0	-	-
**F9**	Uncrosslinked HK loaded PMT–ALG/LF–RST	389.7 ± 1.5	0.423	−44.7 ± 0.32	16/8.33	64.0	12/6.18	80.0	14/7.21	93.3
**F10**	Crosslinked HK-loaded PMT–ALG/LF–RST	258.7 ± 0.95	0.342	−45.3 ± 0.47	16/7.05	64.0	12/5.24	80.0	14/6.11	93.3
**F11**	HK-loaded ALG/LF	278.9 ± 1.7	0.367	−47.4 ± 0.68	-	-	-		10/6.25	66.7

**Table 2:** Effect of genipin amounts on zeta potential, PDI and particle size of nanohybrids F10 (*n* = 3).

Amount of Genipin (mg)	Genipin:F9 wt. Ratio	Particle Size (nm)	PDI
10	1:19.4	389.0 ± 0.30	0.450
20	1:9.7	320.0 ± 0.60	0.410
30	1:6.46	290.0 ± 0.87	0.360
35	1:5.54	258.7 ± 0.95	0.342
50	1:3.88	400.0 ± 1.20	0.420

The amount of HK-loaded PMT–ALG/LF–RST NHs **F9** used is 194 mg.

**Table 3:** Freeze-drying effect on the PS, zeta potential and yield of **F9** and **F10** NHs (*n* = 3).

Formula	Yield (% *w*/*w*)	PS (nm)		RI * (Sf/Si)	ζ-Potential (mV)		PDI	
		Before	After		Before	After	Before	After
HK-loaded PMT–ALG/LF–RST NHs **F9**	92.3%	389.7 ± 0.5	380.0 ± 1.2	0.975	−44.7 ± 1.3	−45.0 ± 0.6	0.423	0.483
Crosslinked HK-loaded PMT–ALG/LF–RST NHs **F10**	94.4%	258.7 ± 0.9	250.0 ± 0.8	0.966	−45.3 ± 0.5	−46.1 ± 0.7	0.342	0.369

* RI: Redispersibility index (Final particle size/Initial particle size).

**Table 4:** CI, IC_50_ and DRI values of free drugs in comparison to the synthesized NHs against MCF-7 breast cancer cells after 24 h at concentrations of 0–100 μM.

Compound	CI Value	Total IC_50_ of Combination	Dose PMT	Dose RST	Dose HK	DRI of PMT	DRI of RST	DRI of HK
HK RST PMT	-	54.95	-	-	-	-	-	-
	-	27.93	-	-	-	-	-	-
	-	19.22	-	-	-	-	-	-
RST/HK PMT/RST	0.691	23.31	-	14.52	9.08	-	1.93	5.81
	0.672	16.31	5.46	10.93	-	3.55	2.56	-
PMT/HK PMT/RST/HK HK-loaded ALG/LF–RST NHs **F4** HK-loaded PMT–ALG/LF NHs **F7**	0.481	14.18	6.40	-	8.00	3.03	-	6.58
	0.286	7.97	1.96	3.91	2.44	9.94	7.15	21.59
	0.142	4.90	-	2.99	1.86	-	9.36	28.26
	0.114	3.50	1.52	-	1.91	12.73	-	27.66
PMT–ALG/LF–RST NHs **F8** HK-loaded PMT–ALG/LF–RST NHs **F9**	0.149	3.65	1.21	2.43	-	16.00	11.51	-
	0.055	1.63	0.38	0.76	0.47	51.22	36.86	111.30
Crosslinked HK-loaded PMT–ALG/LF–RST NHs **F10**	0.033	0.94	0.23	0.46	0.29	84.84	61.04	184.30

## Data Availability

Not applicable.

## Supplementary Materials

The following supporting information can be downloaded at: https://www.mdpi.com/article/10.3390/pharmaceutics14112404/s1, Physicochemical characterization of crosslinked HK-loaded PMT–ALG/LF–RST NHs. In vitro cytotoxicity study and in vitro cellular uptake. In vivo studies. Figure S1: Schematic diagram showing the expected synergy between PMT, HK and RST.; Figure S2: HPLC chromatogram of PMT, RST and HK.; Figure S3: Standard calibration curves of PMT, RST and HK in methanol.; Table S1: List of antibodies, sources, working dilutions, and methods for antigen retrieval.; Table S2: Gradient elution method for quantification of PMT, RST and HK.; Table S3: Retention time, peak performance parameters and wavelength for PMT, RST and HK. References [71,72,73,74,75,76,77,78,79,80,81,82,83,84,85,86] are cited in the supplementary materials.
