# Supplementary material for: Sequential Delivery of Novel Triple Drug Combination via Crosslinked Alginate/Lactoferrin Nanohybrids for Enhanced Breast Cancer Treatment
Source: Pharmaceutics. 2022 Nov 8;14(11):2404. doi: 10.3390/pharmaceutics14112404 (PMC9693489; doi:10.3390/pharmaceutics14112404)
Supplement: Supplementary file 1 [file pharmaceutics-14-02404-s001.zip › pharmaceutics-1986467-supplementary.pdf]

Supporting Information

Sequential delivery of novel triple drug combination *via* crosslinked alginate/lactoferrin nanohybrids for enhanced breast cancer treatment

Introduction:

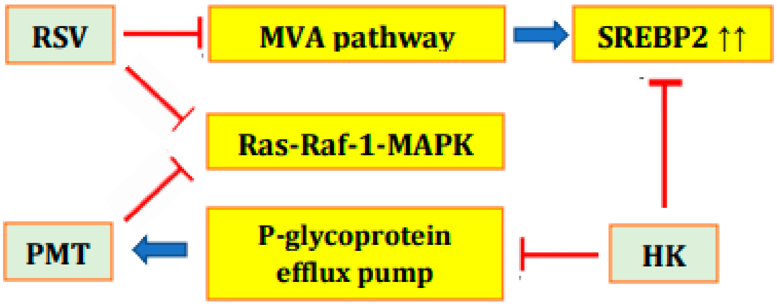

Figure S1: Schematic diagram showing the expected synergy between PMT, HK and RST.

1.Experimental

1.1. Materials

Pemetrexed disodium (PMT), Honokiol (HK), Rosuvastatin calcium (RST) and genipin were purchased from Xi'an Natural Field Bio-Technique Co., Ltd. (China).Sodium Alginate (ALG), lactoferrin (LF), *N, N'*-diisopropylcarbodiimide (DIC),1-ethyl-3-(3dimethylamino) propylcarbodiimide hydrochloride (EDC.HCl), sodium chloride (NaCL), disodium hydrogen phosphate (Na_2HPO_4), potassium dihydrogen orthophosphate (KH_2PO_4), (3-(4,5-dimethylthiazolyl-2)-2,5diphenyl tetrazolium bromide (MTT), fetal bovine serum (FBS), TritonX100, ethyl cyano(hydroxyimino) acetate (Oxyma), Rhodamine, and dimethyl sulfoxide (DMSO) were purchased from Sigma-Aldrich (St. Louis, USA). Methanol and acetonitrile HPLC grade were purchased from Fisher Scientific (Pittsburgh, PA).Human breast adenocarcinoma cell line (MCF7) was purchased from the American Type Culture Collection (ATCC). Absolute ethanol, methanol and orthophosphoric acid were purchased from ADWIC, El-Nasr Pharmaceutical Chemicals Co. (Cairo, Egypt). Potassium salt of ethyl (hydroxyimino) cyanoacetate (K-Oxyma) was prepared in our lab.

1.2. Preparation of Alginate/lactoferrin Nanohybrids Loaded with Honokiol (HK-loaded ALG/LF NHs) F11

Sodium alginate (ALG, 0.05 g) was dissolved in 7 mL double filtrated distilled H_2O. The activation of the carboxylic group of ALG was carried out by *the in-situ* addition of 0.009 g (0.05 mmol) K. Oxyma and 0.01 g (0.05 mmol) EDC. HCl at RT under constant stirring for 5 min. An aqueous solution (5 mL) of lactoferrin (0.10 g, 0.00125 mmol) was added dropwise to the reaction mixture, which was stirred for 24 h at RT. The resultant ALG/LF NHs were then purified by dialysis against double filtrated distilled water. The solvent evaporation method was adopted for the physical loading of 0.015 g of HK in 0.3 mL ethanol into the core of ALG/LF nanohybrids.

1.3. Physicochemical characterization of crosslinked HK-loaded PMT-ALG/LF-RST NHs F10

1.3.1. Drug content:

The conjugation efficiency of RST and PMT in the NHs were determined indirectly from the difference between the amount of free drug in the dialysate and the total initial amount of drug used. After 24 h dialysis, the dialysate was analyzed for the drug content using our developed HPLC method for the determination of RST and PMT. The conjugation efficiency (CE %) of PMT and RST was calculated according to the following equation:

$$\% \text{ CE} = \frac{initial\ drug - free\ drug}{initial\ drug} \times 100 \qquad \text{Eq. 1}$$

The entrapment efficiency of HK in the NHs was determined indirectly. After centrifugation step, the precipitated drug was obtained, dissolved and analyzed using our developed HPLC method for the determination of HK. The entrapment efficiency (EE) of HK was calculated according to the following equation:

$$\% \text{ EE} = \frac{total\ drug - free\ drug}{total\ drug} \times 100 \qquad \text{Eq.2}$$

The percentage drug loading of PMT, RST and HK in the NHs was calculated according to the following equation:

$$\%DL = \frac{Drug\ content}{total\ formula\ wt} \times 100 \qquad \text{Eq.3}$$

Each result is the mean of at least three separate experiments.

1.3.2. Particle size and zeta potential:

The PS of the prepared formulae were measured by DLS with a NanoZS/ZEN3600 Zetasizer. The PS was measured with the non-invasive backscattering technology at a detection angle of 173° after dilution with purified water to an appropriate concentration [71] .All the DLS measurements were performed at 25.0±0.1°C for three repeated measurements. To measure the zeta potential of the prepared NHs; each diluted NHs suspension (1 mL) was put in a universal folded capillary cell equipped with platinum electrodes. The zeta potential was calculated by the Dispersion Technology Software provided by Malvern.

1.4. Proton nuclear magnetic resonance (^{1}H-NMR) spectroscopy:

Nuclear magnetic resonance (NMR) spectra (1H-NMR) were recorded on JEOL 500 MHz spectrometers at ambient temperature. Chemical shifts are reported in parts per million (ppm) and are referenced relative to residual solvent (e.g., DMSO at δ 2.50 ppm for DMSO-d6).

1.5. Solid state characterization

1.5.1 Fourier transform infrared (FT-IR) spectroscopy:

The Fourier transform infrared (FT-IR) spectra of LF, ALG, genipin, LF-RST **F2**, ALG-PMT **F5,** PMT-ALG/LF-RST NHs **F8,** uncrosslinked HK-loaded PMT-ALG/LF-RST NHs **F9,** and crosslinked HK-loaded PMT-ALG/LF-RST NHs **F10** were recorded using FT-IR spectrometer. Samples were finely ground with IR grade dry potassium bromide then pressed into pellets. The spectra were recorded in the range of 4000 to 450 cm-1 at ambient temperature [72].

1.5.2. Differential scanning calorimetry (DSC):

DSC thermograms were recorded for PMT, HK, PMT-ALG/LF-RST NHs **F8,** uncrosslinked HK/PMT-ALG/LF-RST NHs **F9** using a differential scanning calorimeter. Each sample (5 mg) was weighed precisely, placed onto flat bottomed aluminum pan and scanned between 50-500°C with a constant heating rate of 10°C/min in presence of nitrogen atmosphere (flow rate 10 mL/min) [73].

1.6. Morphological analysis:

The morphology of crosslinked HK-loaded PMT-ALG/LF-RST NHs **F10** were examined using transmission electron microscope (TEM) at an accelerating voltage of 80 KV. After sample dilution with water, a sample drop was placed on the copper grid. The excess was drawn off with a filter paper. Samples were subsequently stained with uranyl acetate solution for 30 seconds and then naturally dried before examination [74].

1.7. Physical stability:

The physical stability of the NHs suspension of **F9** and **F10** was monitored with time. For that, aliquots of the NHs were stored in sealed tubes at 4°C. Particle size, PDI and zeta potential of the NHs were monitored at different time points for a period of 3 months [75].

1.8. Freeze-drying and re-dispersibility:

An aliquot of 4 mL of each **F9** and **F10** NHs suspension was transferred into 10 mL glass vials and frozen at 80°C then lyophilized for 48 h. **F9** and F10 were then reconstituted by adding 4 mL of ultrapure water to the lyophilized powder followed by gentle agitation. The reconstituted **F9** and **F10** NHs were evaluated for PS, PDI, zeta -potential and redispersibility index.

1.9. *Invitro* drug release:

The *invitro* release of RST, PMT and HK from **F9** and **F10** NHs and the free drug solutions were investigated using dialysis membrane method [76]. About 3 mL of **F9** NHs colloidal suspension (equiv. to 2.4 mg HK ,1.26 mg RST and 1.5 mg PMT) and 4 mL of **F10** NHs colloidal suspension (equiv. to 2.7 mg HK, 1.4 mg RST and 1.7 mg PMT) were transferred into dialysis bags. The bags were suspended separately in 100 mL phosphate buffered saline (pH 4, 5.5 and 7.4) containing 0.5% SLS to maintain sink conditions and maintained at 37ºC±0.5 in a shaking water bath at 100 rpm under dark conditions. At designated time intervals, 2 mL samples of the release medium were withdrawn followed by compensation with the same volume of fresh release medium. All samples were run in triplicates and filtered through a 0.45 μm membrane filter, then the amounts of HK, RST and PMT released were analyzed by HPLC. The cumulative amounts of drug released over the time period were plotted against the time (h).

1.10. *Invitro* hemolysis:

The Hemolytic activity for **F9** and **F10** NHs was evaluated by *In vitro* hemolysis test by determining hemoglobin release from erythrocyte after incubation with different concentrations of the formula. Briefly, rat blood samples were collected from retro-orbital plexus into test tubes containing ethylene diamine tetra acetic acid (EDTA), centrifuged at 2000 rpm for 15 mins. Supernatant was discarded and sedimented RBCs were collected and washed twice with normal saline (0.9% w/v). The collected RBCs were then diluted with normal saline to obtain 10 mL of a final concentration of 2% v/v RBCs suspension. About 2 mL of the RBC suspension was incubated separately with 2 mL of each the four concentrations of each formula (0.25mg\mL, 0.5mg\mL, 1mg\mL, 2mg/mL) at 37°C±0.5 with a mild shaking in the shaking water bath at 100 rpm. After 1 h, the samples were centrifuged at 3000 rpm for 5 min and supernatants were collected for hemoglobin content quantification by spectrophotometric analysis at λmax 545 nm, against a negative control. The negative control (0% lysis) was prepared by mixing 2 mL of the RBC suspension with 2 mL of saline, while 100% hemolysis was induced by treating the RBCs with 1% w/v Triton X100 instead of normal saline and used as a positive control. The hemolytic rates of the samples were calculated as the following equation:

Hemolytic rate (%) = $(A_t - A_{nc}) \backslash (A_{pc} - A_{nc})$ X 100% Eq. 4

Where At represents the absorbance value of test sample, Anc and Apc represent the absorbance value of negative and positive controls, respectively [77]. All procedures were performed according to by the optimal use of experimental animals at the Pharmaceutical and Fermentation Industries Development Center of the General Authority of the City of Scientific Research and Technological Applications (SRTA-city), Alexandria, Egypt, and in accordance with regulations of the National Research Council 's guide for the care and use of laboratory animals, IACUCs / IACUC # 73-3N-1022.

1.11. *Invitro* serum stability:

In order to mimic blood circulation conditions, **F9** and **F10** NHs were incubated in a shaking water bath at 37°C under mild stirring with an equal volume of 10% w/v fetal bovine serum (FBS) for 6 h. At each time interval (0, 1, 2,3, 4 and 6 h), 50 µL of the mixture was withdrawn then diluted in distilled water (1:50 v/v) to be assessed for their PS and PDI, using dynamic light scattering (DLS) technique [78].

2. ***In vitro*** **cytotoxicity study:**

The cytotoxicity of the blank ALG/LF NHs **F1**, free HK, free RST, free PMT, free combination therapies (RST/HK, PMT/RST, PMT/HK) and free combination therapy (PMT/RST/HK), dual drug loaded NHs (HK loaded-ALG/LF-RST **F4**, PMT-ALG/LF-RST **F8** and HK loaded-PMT-ALG/LF **F7**, uncrosslinked HK-loaded PMT-ALG-LF-RST NHs **F9** and crosslinked HK-loaded PMT-ALG-LF-RST NHs **F10** on MCF-7 breast cancer cells were evaluated by the MTT (3-(4,5-dimethylthiazol-2-yl)-2,5-diphenyltetrazolium bromide) colorimetric assay. The cells were maintained in DMEM containing 10% FBS in a CO2 incubator (5% CO2 at 37°C). Cells were seeded at a density of 5 × 103 cell/well in a 96-well plate containing 100 μL of DMEM enriched and allowed to adhere to the plate overnight. The medium was replaced by fresh medium containing different concentrations of the drugs either as a solution or the prepared NHs, and incubated for another 24 h. A stock solution of free drugs and its combination were dissolved individually in water or DMSO using concentration of 1 mg/mL and stored at -70ºC. Concentration of DMSO in the medium was kept <0.1%. The culture medium was then replaced with 100 μL of MTT solution (0.5 mg/mL in DMEM) then incubated for further 4 h at 37°C in the dark. After removal of MTT solution by centrifugation at 2000 rpm for 10 min, 100 μL of DMSO were added to the wells to dissolve MTT-formazan crystals formed after internalization of MTT by live cells and maintained in agitation for 15 min. Absorbance of the converted dye was measured at a wavelength of 570 nm with background subtraction at 690 nm using a microplate reader (Model 550, Bio-Rad, USA). The relative cell viability was expressed as a percentage of the untreated control wells. The inhibitory concentration (IC50) values were determined using Origin 8.0 (Origin Lab, Northampton, MA) according to the fitted data [79]. The percentage of cell viability related to control cells incubated with culture medium only, was determined by the following equation:

$$\%\text{Cell viability} = [A(\text{test}) / A(\text{control})] \times 100 \qquad \text{Eq.5}$$

Where A (test) is the absorbance obtained from the test sample and A (control) is the absorbance obtained from untreated cells (incubated with medium only). The latter reading was assumed to correspond to 100% cell viability. The MTT assay for each concentration was performed in triplicate. The 50% cell cytotoxic concentration (IC_{50}), the concentration required to kill 50% of the cells, was estimated.

3. *In vitro* cellular uptake study:

Stock solution of rhodamine B isothiocyanate (RBITC) solution was prepared in DMSO (5mg/mL) and maintained in the dark. 0.2 mL of the stock RBITC solution was added to the prepared uncrosslinked PMT-ALG/LF-RST **F8** and crosslinked PMT-ALG/LS-RST NHs solution. The mixture was allowed to react overnight in dark conditions followed by purification by dialysis method against deionized water for another 24 h to measure the amount of the unreacted RBITC using UV. The labeled NHs were freeze-dried for further use and characterized by measuring the PS and zeta potential to monitor the effect of the incorporation of RBITC. MCF-7 cells were seeded on glass coverslips placed in six-well culture plates overnight at a density of 1.8×10^5 cells. The medium was then replaced with fresh medium adjusted to pH 7.4, which mixed with the labelled uncrosslinked **F8** and crosslinked **F8** NHs. After incubation, the medium was removed, and the cells were washed three times with cold PBS to terminate the cell uptake. After that, the cells were fixed with 4% paraformaldehyde solution and then the cover slips were then mounted onto microscope slides using 2-(4-ethoxyphenyl)-6-[6-(4-methylpiperazin-1-yl)-1*H*-benzimidazol-2yl]-1*H*-benzimidazole (Hoechst) containing (DPX) as mounting medium. The regular DMEM was applied as the blank control. In order to assess the competitive uptake efficacy, the cells were pre-incubated with 50 µg/mL of excessive free lactoferrin for 30 min at 37°C±0.5.

3.1. Confocal Microscopy Study:

Imaging analysis was performed via confocal laser scanning microscopy using excitation wavelength of 488 nm, and emission wavelength of 530 nm. Cellular uptake was quantitatively determined by flow cytometer.

4. *In vivo* studies

4.1. Animals

In vivo study was carried out on BALB C female mice (7-8 weeks, 25 ± 5 g) housed in stainless steel mesh cages in 7 groups each of seven mice, under standard conditions of light illumination, relative humidity, and temperature, and they had free access to standard laboratory food and water throughout the study. All animal experimental procedures were performed according to a protocol approved by the Animal Care and Use Committee of the City of Scientific Research and Technological Applications (SRTA-city), Alexandria, Egypt, and in accordance with regulations

of the National Re-search Council's guide for the care and use of laboratory animals, IACUCs / IACUC # 73-3N-1022.

4.2. Development of tumor model

Female BALB/C mice (7-8 weeks of age) were housed in a pathogen-free environment at 7 mice/cage. They were supplied with autoclaved and non-fluorescent mouse chow and water. Ehrlich ascites tumor (EAT) cells, supplied from National Institute of Cancer, Egypt, were collected from the ascitic fluid of BALB/C mice harbouring 8–10 days old ascitic tumor. Approximately, 10^7 of EAT cells suspended in PBS were injected into the left side of the mammary fat pad of BALB/C female mice [80]. Tumor growth was monitored daily until its volume reached 100 mm^3. Tumor volume was determined by measuring both perpendicular diameters of the tumor using a micrometer according to the following equation [81].

$$Tumor\ volume = \frac{L \times W \times W}{2} \qquad (7)$$

where W is tumor width, L is tumor length.

4.3 *In vivo* anti-tumor efficacy

To evaluate the *in vivo* anti-tumor efficacy, animals were randomly divided into 7 groups (7 mice per group). The groups included negative control (healthy mice injected with saline), positive control (inoculated with EAT but left untreated), free PMT, free HK solution, free RST, free (PMT/RST/HK) combination and crosslinked HK- loaded PMT-ALG/LF-RST NHs **F10** treated-groups. The treatment period was started when the tumor size reached to about 100 mm^3 in almost groups (within 10 days). The tumor-bearing mice were injected i.v through the tail vein with free drugs or NHs equivalent to 4 mg /kg, 10 mg/kg RST and 2.5 mg /kg HK three times per week for three weeks [38,82,83]. At the end of treatment period, all mice groups were sacrificed by cervical dislocation after anaesthetizing by ether inhalation. Tumors were surgically removed and the weights were determined. Tumors were flushed with phosphate buffer saline (PBS, pH 7.4). Each excised tumor was divided into 2 parts. The first part was fixed in 10% neutral buffered formalin for 48 hr and embedded in paraffin blocks for histopathological examination. The other part was homogenized using PBS, and aliquots were preserved at -80°C for further determination of markers of anti-tumor activity. Also, liver and kidney specimens were surgically removed and

flushed with phosphate buffer saline (PBS, pH 7.4) then fixed in 10% neutral buffered formalin for 48 h and embedded in paraffin blocks for histopathological examination.

4.4. Histopathological study and scoring system

The fixed specimens were processed by the conventional paraffin embedding technique including the dehydration through ascending grades of ethanol, clearing in three changes of xylene and melted paraffin ended by embedding in paraffin wax at 65°C. Four µm thick sections were stained by Hematoxylin and Eosin (H and E) as previously described by Bancroft and Layton [84]. In each excised tumor, the semi-quantitative scoring of necrosis was assessed in ten random fields (HPF; ×40). Scoring scale was ranged from 1 to 4 based on the following criterion: Score 1; about 10% necrosis was shown in poorly differentiated neoplasm, score 2; about 25% necrosis was shown in poorly differentiated neoplasm, score 3; about 35% necrosis was shown in poorly differentiated neoplasm,4; more than 50% necrosis was shown in poorly differentiated neoplasm. The mean value of all scores (10 fields/tumor sample) was recorded, analyzed, and expressed as Mean of necrosis scale ± S.E.

4.5 Immunohistochemical study

Antibodies, sources, working dilutions, and methods for antigen retrieval were listed in (Tabel.1). The immunohistochemical technique in liver sections was investigated according to the method described by Noreldin*, et al.* [85]. Briefly, four-µm-thick paraffin sections were prepared and deparaffinized by xylene then rehydrated in graded alcohols and washed by distilled water. After washing with distilled water**,** endogenous peroxidase was deactivated by 3% H_2O_2 in absolute methanol for 5 min at 4°C. After washing with PBS, the nonspecific reaction was blocked with 10% normal blocking serum for 60 min. at room temperature. Then, the primary antibodies were incubated at 4°C overnight. After washing with PBS, the sections were incubated with biotin-conjugated goat anti-rabbit IgG antiserum (Histofine kit, Nichirei Corporation) for 60 min. then washed in PBS, followed by incubation with streptavidin-peroxidase conjugate (Histofine kit, Nichirei Corporation) for 30 min. The streptavidin-biotin complex was visualized with 3, 3′-diaminobenzidine tetrahydrochloride (DAB)-H_2O_2 solution, pH 7.0, for 3 min. Then sections were washed in distilled water and Mayer's hematoxylin and used as a counterstain. For the quantitative histomorphometric analysis, original micrographs were captured from the immunostained slides (10 random fields from each section, x400 for caspase 3 and Ki67 by a digital camera (Leica EC3, Leica, Germany) connected to a microscope (Leica DM500). The immune positive cells were

counted in each examined fields by the aid of manual computer-assisted cell counting (Image J plug-in -cell_counter.jar) using ImageJ software (v1.46r, NIH, Bethesda, MD, USA) [86].

Table S1. List of antibodies, sources, working dilutions, and methods for antigen retrieval.

Antibody	Source	Dilution	Antigen retrieval	Heating condition
Rabbit polyclonal anti-Caspase 3	(9662, Cell Signaling Technology, Danvers, Ma, USA)	1:300	10 mM citrate buffer (pH 6.0)	105°C, 20 min
Rabbit polyclonal anti-Ki67	(GTX20833, GeneTex,CA, USA)	1:150	10 mM citrate buffer (pH 6.0)	105°C, 20 min

5. Statistics:

For all *in vitro* characterization, all measurements were carried out in triplicate and values are presented as the mean ± S.D. For comparison of mean values between groups, Analysis of Variance test (ANOVA) and Tukey's Multiple Comparison test were used. The difference was considered significant when P-values < 0.05.

Results and Discussion

1. **HPLC assay of PMT, RST and HK:**

The HPLC analysis was carried out with an Inertsil® ODS-3 reversed-phase column (250 × 4.6 mm, 5 µm, GL Sciences Inc.). The column was maintained at room temperature. For chromatographic elution, the injection volume was 5 µL. A step gradient method was utilized

Table S2: Gradient elution method for quantification of PMT, RST and HK.

Step	Time	Flow rate (mL/min)	Mobile phase A% (methanol)	Mobile phase B% (phosphate buffer saline, pH 6 adjusted using orthophosphoric acid)
1	0.0	0.7	80	20
2	6.0	0.7	80	20

3	7.0	1.0	80	20
4	13.0	1.0	80	20
5	13.5	0.7	80	20

Running the HPLC analysis, A good linearity was shown by the calibration curve obtained from 2 to 20 µg/mL of PMT, RST and HK concentration

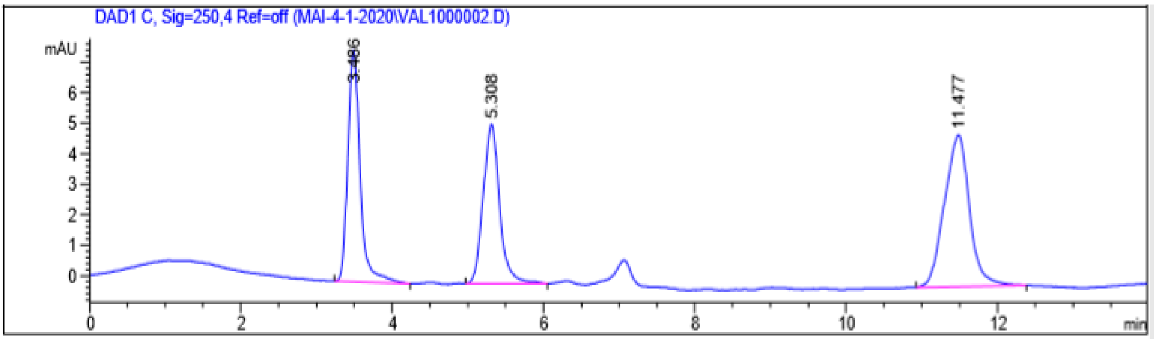

Figure S2: HPLC chromatogram of PMT, RST and HK.

Table S3: Retention time, peak performance parameters and wavelength for PMT, RST and HK.

Drug	Retention time(min)	Tailing factor	Wavelength(nm)
PMT	3.48	0.799	225
RST	5.30	0.967	243
HK	11.40	1.100	294

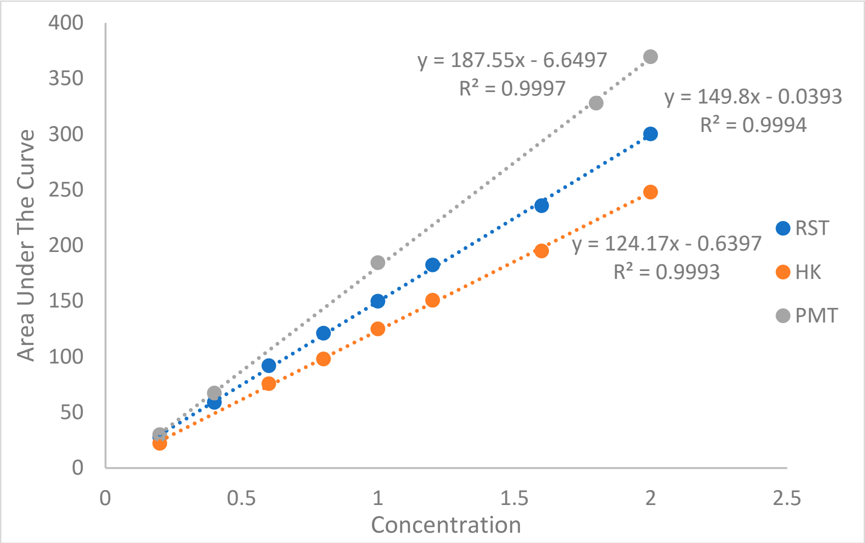

Figure S3: Standard calibration curves of PMT, RST and HK in methanol

References

71. Elzoghby, A.O.; Helmy, M.W.; Samy, W.M.; Elgindy, N.A. Micellar Delivery of Flutamide Via Milk Protein Nanovehicles Enhances its Anti-Tumor Efficacy in Androgen-Dependent Prostate Cancer Rat Model. *Pharm. Res.* **2013**, *30*, 2654-2663, doi:10.1007/s11095-013-1091-7.
72. Abdelmoneem, M.A.; Mahmoud, M.; Zaky, A.; Helmy, M.W.; Sallam, M.; Fang, J.Y.; Elkhodairy, K.A.; Elzoghby, A.O. Decorating protein nanospheres with lactoferrin enhances oral COX-2 inhibitor/herbal therapy of hepatocellular carcinoma. *Nanomedicine (Lond)* **2018**, *13*, 2377-2395, doi:10.2217/nnm-2018-0134.
73. Elzoghby, A.O.; Vranic, B.Z.; Samy, W.M.; Elgindy, N.A. Swellable floating tablet based on spray-dried casein nanoparticles: Near-infrared spectral characterization and floating matrix evaluation. *Int J Pharm* **2015**, *491*, 113-122, doi:https://doi.org/10.1016/j.ijpharm.2015.06.015.
74. Elzoghby, A.O.; Saad, N.I.; Helmy, M.W.; Samy, W.M.; Elgindy, N.A. Ionically-crosslinked milk protein nanoparticles as flutamide carriers for effective anticancer activity in prostate cancer-bearing rats. *Eur J Pharm Biopharm* **2013**, *85*,451,doi:https://doi.org/10.1016/j.ejpb.2013.07.003.
75. Fang, M.; Jin, Y.; Bao, W.; Gao, H.; Xu, M.; Wang, D.; Wang, X.; Yao, P.; Liu, L. In vitro characterization and in vivo evaluation of nanostructured lipid curcumin carriers for intragastric administration. *Int J Nanomedicine* **2012**, *7*, 5395-5404, doi:10.2147/IJN.S36257.
76. Elgindy, N.; Elkhodairy, K.; Molokhia, A.; Elzoghby, A. Biopolymeric Nanoparticles for Oral Protein Delivery: Design and In Vitro Evaluation. *J Nanomed Nanotechnol* **2011**, *2*, doi:10.4172/2157-7439.1000110.
77. Shim, M.S.; Lee, H.T.; Shim, W.S.; Park, I.; Lee, H.; Chang, T.; Kim, S.W.; Lee, D.S. Poly(D,L-lactic acid-co-glycolic acid)-b-poly(ethylene glycol)-b-poly (D,L-lactic acid-co-glycolic acid) triblock copolymer and thermoreversible phase transition in water. *J. Biomed. Mater. Res.* **2002**, *61*, 188-196, doi:10.1002/jbm.10164.
78. Wolfram, J.; Suri, K.; Huang, Y.; Molinaro, R.; Borsoi, C.; Scott, B.; Boom, K.; Paolino, D.; Fresta, M.; Wang, J.; et al. Evaluation of anticancer activity of celastrol liposomes in prostate cancer cells. *J Microencapsul* **2014**, *31*, 501-507, doi:10.3109/02652048.2013.879932.
79. Nie, Y.; Ji, L.; Ding, H.; Xie, L.; Li, L.; He, B.; Wu, Y.; Gu, Z. Cholesterol derivatives based charged liposomes for doxorubicin delivery: preparation, in vitro and in vivo characterization. *Theranostics* **2012**, *2*, 1092-1103, doi:10.7150/thno.4949.
80. Kujala, E.; Toivonen, P. Calculated tumour volume as a prognostic parameter for survival in choroidal melanomas. *Eye* **2006**, *20*, 123-124.
81. Jensen, M.M.; Jørgensen, J.T.; Binderup, T.; Kjær, A. Tumor volume in subcutaneous mouse xenografts measured by microCT is more accurate and reproducible than determined by 18 F-FDG-microPET or external caliper. *BMC Med Imaging* **2008**, *8*, 16.
82. Chiang, C.-K.; Sheu, M.-L.; Hung, K.-Y.; Wu, K.-D.; Liu, S.-H. Honokiol, a small molecular weight natural product, alleviates experimental mesangial proliferative glomerulonephritis. *Kidney Int* **2006**, *70*, 682-689.
83. Radigan, K.A.; Urich, D.; Misharin, A.V.; Chiarella, S.E.; Soberanes, S.; Gonzalez, A.; Perlman, H.; Wunderink, R.G.; Budinger, G.S.; Mutlu, G.M.J.P.o. The effect of rosuvastatin in a murine model of influenza A infection.*PloS one*, **2012**, *7*, e35788.
84 Bancroft, J.D.; Layton, C. The hematoxylin and eosin, connective and mesenchymal tissues with their stains. In *Bancroft s Theory and practice of histological techniques*, 7th ed.; S. Kim suvarna, C.L.a.J.D.B., Ed.; Churchill Livingstone:: Philadelphia 2013; pp. 173-186.

85. Noreldin, A.E.; Elewa, Y.H.A.; Kon, Y.; Warita, K.; Hosaka, Y.Z. Immunohistochemical localization of osteoblast activating peptide in the mouse kidney. *Acta histochemica* **2018**, *120*, 323-328.
86. Schneider, C.A.; Rasband, W.S.; Eliceiri, K.W. NIH Image to ImageJ: 25 years of image analysis. *Nat Methods* **2012**, *9*, 671-675.
